# Engineering an Acyl‐CoA Ligase With Enhanced Activity Toward Synthetic CoA Alternatives

**DOI:** 10.1002/cbic.70385

**Published:** 2026-05-13

**Authors:** Jared R. Cossin, Sarah A. Taboada, Gavin J. Williams

**Affiliations:** ^1^ North Carolina State University Raleigh North Carolina USA; ^2^ Florida State University Tallahassee Florida USA; ^3^ Comparative Medicine Institute Raleigh North Carolina USA

**Keywords:** acyl‐CoA ligases, biocatalysis, biosynthesis, directed evolution, polyketides

## Abstract

Fatty acyl‐coenzyme A (CoA) thioesters are indispensable intermediates in primary metabolism and essential building blocks for biosynthetic pathways yielding fuels, fine chemicals, and pharmaceuticals. However, large‐scale production of acyl‐CoAs is frequently constrained by the intracellular availability and high cost of CoA, motivating the development of alternative strategies for precursor generation. Here, we report the engineering of the acylCoA ligase AcsA_*Pc*_ to preferentially utilize inexpensive, membrane‐permeable synthetic CoA surrogates, with a focus on N‐acetylcysteamine (SNAC). Building on a broad specificity variant (D449E), structure‐guided saturation mutagenesis and random mutagenesis, coupled with high‐throughput colorimetric screening, progressively shifted thiol specificity from CoA to pantetheine and SNAC. The resulting double mutant, F430W/D449E, exhibits a 26‐fold improvement in SNAC utilization relative to wildtype while concomitantly reducing CoA activity. Kinetic analyses reveal that these gains arise from increased reaction rates with synthetic thiols and altered substrate preferences across a representative acid panel. The functional relevance of AcsA‐generated acyl‐SNACs was demonstrated in a reconstituted polyketide system, where enhanced availability of SNAC‐linked starter units drove an ∼8‐fold increase in pyrone formation. This work establishes AcsA as a tunable platform for orthogonal generation of polyketide precursors and a general framework for alleviating CoA‐dependent biosynthetic bottlenecks.

## Graphical abstract

Engineering an acyl‐CoA ligase shifts thiol specificity toward inexpensive synthetic thiols such as N‐acetylcysteamine (SNAC), enabling enzymatic generation of acyl‐SNAC starter units that support polyketide biosynthesis.

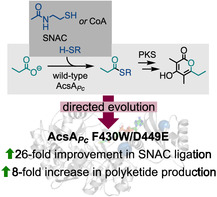

## 1 Introduction

**FIGURE 1:**
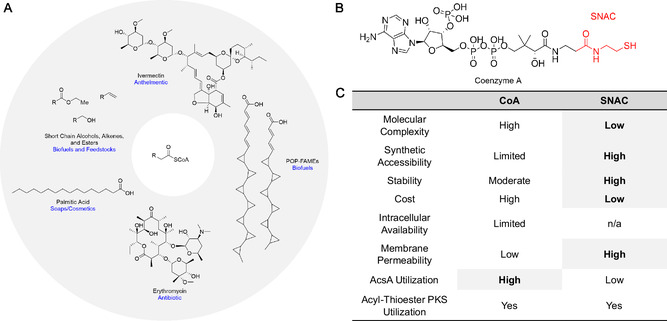
SNAC as a minimal functional surrogate for CoA in polyketide biosynthesis. (A) Representative polyketide‐ and lipid‐derived products illustrating the central role of acyl‐thioester starter units in diverse biosynthetic and industrial contexts. The acyl‐SCoA motif common to these pathways is shown at the center. (B) Structural comparison of acyl‐CoA and acyl‐SNAC highlighting the shared structure (red), which constitutes the chemically essential handle for acyl transfer during PKS catalysis. (C) Comparison of physicochemical and practical properties of acyl‐CoA and acyl‐SNAC relevant to in vitro PKS studies.

Fatty acyl‐coenzyme A (CoA) thioesters occupy a central position in cellular metabolism, functioning as activated electrophiles that participate in fatty acid biosynthesis, β‐oxidation, and the assembly of complex natural products such as polyketides [1] and nonribosomal peptides (Figure 1) [2]. Beyond their native biological roles, acyl‐CoAs have emerged as valuable intermediates for the sustainable production of biofuels [3, 4], green chemicals [5], and pharmaceuticals [3, 6]. As interest in biocatalytic and fermentation‐based manufacturing continues to grow, so too does the demand for efficient and economical routes to these activated precursors.

Despite their ubiquity, the production of acyl‐CoAs at preparative scales remains a persistent challenge. In vivo, product titers are often limited by intracellular CoA availability and the metabolic burden imposed by diverting CoA from essential housekeeping functions [7, 8]. In vitro and semi‐synthetic approaches face a different but equally restrictive barrier: CoA is expensive, typically costing several thousand dollars per gram, rendering large‐scale or high‐throughput applications impractical [9, 10]. Together, these constraints motivate the exploration of alternative thiols that can substitute for CoA while retaining compatibility with downstream biosynthetic enzymes.

Among the synthetic CoA surrogates that have been explored, (*R*)‐pantetheine and *N*‐acetylcysteamine (SNAC) are particularly attractive. Both share structural features with the terminal portion of CoA and have been shown to support catalysis by a variety of acyltransferases and polyketide synthase (PKS) domains [11, 12, 13, 14]. SNAC, in particular, offers several advantages, including low cost, straightforward synthesis, and membrane permeability, enabling both in vitro and intracellular applications. Numerous studies have demonstrated that SNAC‐linked intermediates can be accepted by downstream enzymes, suggesting that efficient enzymatic access to acyl‐SNACs could provide a powerful strategy for precursor‐directed biosynthesis [11, 12, 13, 14].

Previously, we reported that mutation of Asp449 to Glu in the propionyl‐CoA synthetase AcsA_*Pc*_ broadened the enzyme's carboxylic acid scope and modestly improved its ability to utilize non‐native thiols, including pantetheine and SNAC [15]. Although these activities were low, they suggested that AcsA could be a promising starting point for engineering enhanced specificity toward synthetic CoA analogs. Here, we expand on this observation by systematically evolving AcsA_*Pc*_ toward favoring pantetheine and SNAC over CoA. Through iterative rounds of mutagenesis and screening, we identify key active‐site residues that govern thiol selectivity, characterize the kinetic and substrate‐scope consequences of these mutations, and demonstrate the utility of the engineered enzyme in a reconstituted polyketide biosynthetic context.

## 2 Results and Discussion

### 2.1 Baseline Thiol Specificity of AcsA_*Pc*_ D449E

We began by quantitatively assessing the thiol specificity of the N‐terminally hexa‐His‐tagged fusion protein, AcsA_*Pc*_ D449E. Using propanoic acid as a model carboxylate substrate, in vitro HPLC and/or LC‐MS‐based endpoint assays revealed that D449E retains near‐quantitative conversion with CoA (**1**) (97.7 ± 1.2%) while exhibiting modest but measurable activity with pantetheine (**2**) (12.8 ± 1.3%) and SNAC (**3**) (3.9 ± 0.7%) (Table S1, Supporting Information). In contrast, the wild‐type enzyme showed only trace activity with pantetheine and SNAC (2.2 ± 0.1% and 0.3 ± 0.1%, respectively). Nevertheless, these data establish D449E as a viable evolutionary intermediate, providing both a substantial dynamic range for improvement and a measurable baseline activity with the synthetic thiols of interest.

### 2.2 Structure‐Guided and Random Mutagenesis Strategy

**FIGURE 2:**
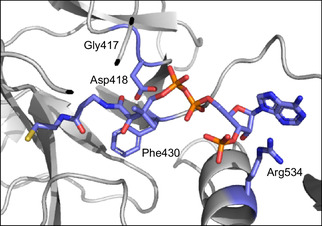
AcsA docking study with various thiol substrates. Docking of CoA (sticks colored by element) within the active site of an AlphaFold predicted structure of AcsA*_Pc_* D449E. Shown are residues (blue sticks) within 5.0 Å of CoA that were selected for mutagenesis.

To identify mutations that further enhance synthetic thiol utilization, we adopted a dual mutagenesis strategy combining structure‐guided saturation mutagenesis with error‐prone PCR. An AlphaFold‐predicted [16] structure of AcsA_*Pc*_ D449E was used to dock CoA (**1**) into the active site, revealing residues within 5 Å of the bound thiol that were likely to influence thiol accommodation (Figure 2). Based on proximity, size, and hydrogen‐bonding potential, Gly417, Asp418, Phe430, and Arg534 were each selected for site‐saturation mutagenesis in D449E. In parallel, to sample non‐obvious positions that might contribute to altered thiol specificity, random mutagenesis was performed using the D449E variant as a template, yielding a library with an average of 1–2 amino acid substitutions per gene.

### 2.3 High‐Throughput Screening and Identification of Beneficial Mutations

As a means of high‐throughput screening, the thiol‐sensitive Ellman's reagent [17, 18] was used to monitor consumption of the thiol substrate via its absorbance detection at 412 nm. Owing to the large size disparity between **1** and **3**, the initial activity with **3** fell outside the screen's limit of detection (data not shown). Therefore, pantetheine (**2**) was used as a surrogate screening substrate, with the expectation that improvement with pantetheine would translate into increased utilization of **3**. Propanoic acid (**4**) was chosen as the acyl donor due to its low cost and high conversion with the D449E parent.

**FIGURE 3:**
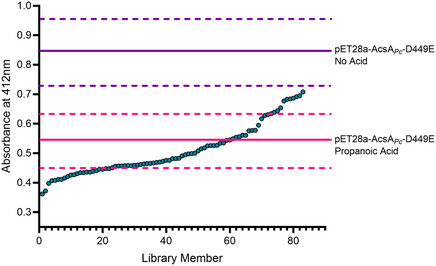
High‐throughput screening of AcsA*_Pc_* D449E mutant libraries. Representative absorbance values at 412 nm for individual mutants from the Phe430X saturation library, plotted in ascending order. Each point corresponds to a single library member assayed using Ellman's reagent with propanoic acid and (*R*)‐pantetheine. The magenta solid line indicates the mean absorbance of the D449E parent (n = 3), with dashed lines denoting ±3 standard deviations. The purple solid and dashed lines represent the mean and ±3 standard deviations of the no‐acid negative control, respectively.

Subsequently, individual colonies from the saturation and random mutagenesis libraries were cultured and expressed in wells of 96‐well microplates. Following growth, cells were lysed in situ for colorimetric screening. Initial hits were defined as those demonstrating an absorbance reading three or more standard deviations below the parent average (Figure 3). To confirm the identification of positive hits, wells containing mutants that led to lower absorbance readings were grown again and assayed in microtiter plates in triplicate.

Screening the saturation mutagenesis and random mutagenesis libraries identified several variants exhibiting significantly reduced absorbance relative to the parent enzyme, indicative of increased thiol utilization. From the saturation mutagenesis libraries, Phe430 yielded two particularly promising single amino acid mutations, F430V and F430W. The random mutagenesis library provided two additional single mutations, L250M and R302S. Rescreening and validation assays established these mutations as reproducible improvements over the D449E background.

### 2.4 Recombination and Evaluation of Mutant Combinations

**FIGURE 4:**
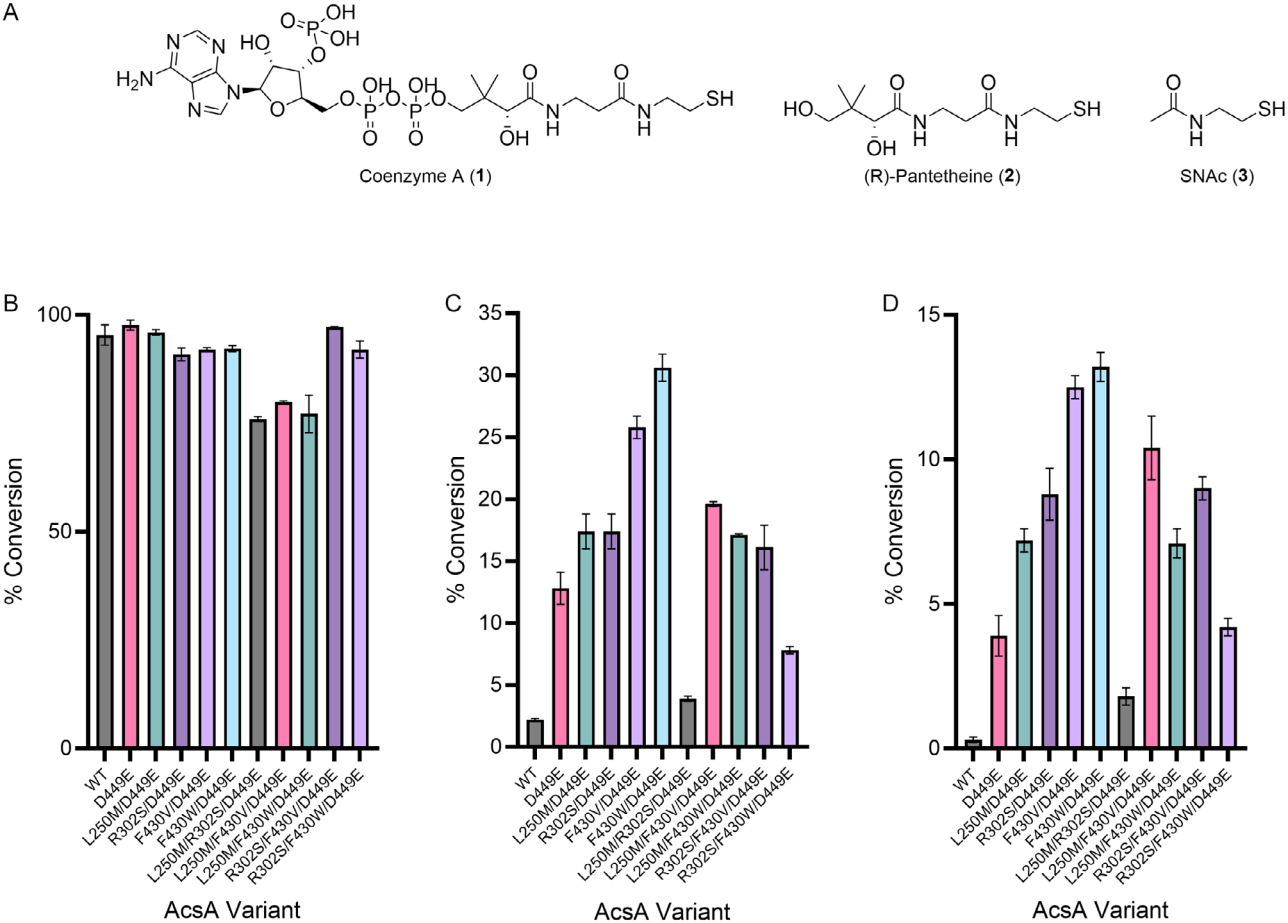
Relative substrate specificity of AcsA*_Pc_* variants determined by one‐hour end‐point assays. (A) Chemical structures of coenzyme A (CoA) and the synthetic thiol alternatives (R)‐pantetheine and *N*‐acetylcysteamine (SNAC) evaluated in this study. (B–D) One‐hour in vitro end‐point assays measuring conversion of carboxylic acid substrate to the corresponding activated thioester by wild‐type AcsA*_Pc_*, D449E, and selected combinatorial variants using (B) CoA, (C) (*R*)‐pantetheine, or (D) SNAC as the thiol substrate. Bars represent mean values from technical replicates (n = 3), and error bars indicate the standard deviation.

To assess whether beneficial mutations could act additively or synergistically, a series of triple mutants was constructed in which the D449E substitution was held constant, and all possible combinations of the remaining mutations were generated by site‐directed mutagenesis, expressed, and purified via metal‐ion affinity chromatography. End‐point assays with propanoic acid (**4**), CoA (**1**), pantetheine (**2**), and SNAC (**3**) (Figure 4A and Table S5, Supporting Information), followed by LCMS analysis, revealed that each of the identified mutants exhibited improved activity with both **2** and **3** relative to the D449E parent (Figure 4C,D). For instance, introduction of the L250M or R302S substitutions resulted in modest enhancements, corresponding to ∼1.3‐ and ∼1.5‐fold increases in conversion with **2** and **3**, respectively (Figure 4C,D). Notably, larger gains were observed for mutations at Phe430. The D449E/F430V variant afforded ∼2.0‐ and ∼2.6‐fold improvements in **2** and **3** utilization, respectively, while the D449E/F430W variant displayed ∼2.4‐ and ∼2.8‐fold higher conversion with **2** and **3**, respectively, relative to D449E. Notably, relative to the wild‐type enzyme, the D449E/F430W mutant exhibits ∼13.7‐ and ∼26‐fold enhancements in activity with **2** and **3**, respectively. Consistent with the docking predictions (Figure 2), these improvements implicate Phe430 as a key determinant of thiol recognition, with the F430W substitution might stabilize the C‐7 ketone of **3** through π‐dipole interactions, whereas the impact of the F430V mutation is more plausibly attributed to altered steric or van der Waals contacts.

In contrast, recombination of beneficial mutations into triple‐mutant variants failed to produce additive or synergistic effects, as judged by the endpoint conversion assays (Figure 4C,D). Triple mutants containing the L250M substitution exhibited reduced conversion with **1** relative to the corresponding double mutants (Figure 4B), accompanied by diminished activity with **2** and **3**. These observations suggest deleterious epistatic interactions that compromise overall catalytic efficiency, underscoring the non‐additive nature of active‐site remodeling in AcsA_*Pc*_.

### 2.5 Initial Reaction Rates and Thiol Preference Shifts

To further dissect the improved SNAC (**3**) utilization, we measured initial reaction rates for representative variants (Table 1 and Figure S3, Supporting Information). While the D449E mutation increased rates for all thiols, introduction of the F430W substitution selectively enhanced rates with pantetheine and SNAC (**3**) while decreasing the rate with CoA (**1**) by 0.73‐fold. This partial inversion of thiol preference is particularly desirable for applications aimed at orthogonal precursor generation, as it reduces competition with endogenous CoA pools.

**TABLE 1:** Initial reaction rates and thiol utilization profiles of AcsA_Pc_ variants.

Variant	CoA, 1		Pantetheine, 2		SNAC, 3	
	Initial Rate^a^	Fold Change^b^	Initial Rate^a^	Fold Change^b^	Initial Rate^a^	Fold Change^b^
WT	12.6 ± 0.3	N.A.^c^	0.10 ± 0.02	N.A.^c^	N.D.^d^	N.A.^c^
D449E	17.4 ± 0.7	1.38	1.1 ± 0.2	11.0	0.27 ± 0.07	9.6 b
F430W/ D449E	9.17 ± 0.57	0.73	2.03 ± 0.14	20	0.72 ± 0.04	26 b

a Initial rate (µmol product sec^−1^) measured over the first 10 min. Rates were determined from linear fits of product formation versus time and are reported as mean ± standard deviation from technical replicates (*n* = 3).

b Fold change in activity relative to the preceding variant generation. The limit of detection value was used for fold‐change calculations where applicable.

c N.A., Not applicable.

d N.D., not detected (limit of detection = 0.028 μmol product s^−1^).

### 2.6 Determining the Acid Profile of AcsA Mutants

**FIGURE 5:**
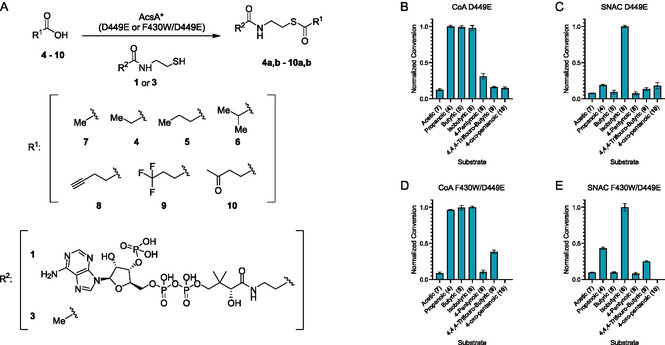
Thiol‐dependent activation of carboxylic acid substrates by engineered AcsA variants and comparative substrate conversion profiles. (A) Reaction scheme illustrating AcsA‐catalyzed activation of carboxylic acid substrates (**4**–**10**) in the presence of either coenzyme A (CoA, **1**) or *N*‐acetylcysteamine (SNAC, **3**). The identity of the activated product is determined by the thiol source: CoA yields the corresponding acyl‐CoA thioesters (**4a**–**10a**), whereas SNAC yields the corresponding acyl‐SNAC thioesters (**4b**–**10b**). (B,C) Normalized conversion of carboxylic acid substrates to (B) acyl‐CoAs or (C) acyl‐SNACs by the AcsA D449E variant. (D,E) Normalized conversion of the same substrate panel to (D) acyl‐CoAs or (E) acyl‐SNACs by the AcsA F430W/D449E variant. Conversion values were normalized to the highest‐converting substrate within each enzyme/thiol pair. Error bars represent the standard deviation of technical replicates (n = 3).

To evaluate whether the introduced mutations alter the carboxylic acid scope of the enzyme, a representative panel of substrates spanning straight‐chain (**4**‐**5**, **7**) and branched (**6**) aliphatic, unsaturated (**8**), halogenated (**9**) and oxidized (**10**) acids were assayed with the D449E and F430W/D449E variants in the presence of CoA (**1**) (Figure 5A andTable S6, Supporting Information). The D449E mutant was selected as the reference enzyme because it served as the starting point for the mutagenesis campaign and had previously been shown to exhibit broadened substrate promiscuity. Consistent with a report [15], D449E converted all tested acids, with propanoic (**4**), butyric (**5**), and isobutyric (**6**) acids emerging as the most favored substrates (Figure 5B and Table S6, Supporting Information). While the F430W/D449E variant retained comparable activity toward these preferred acids (Figure 5B), it exhibited reduced conversion of acetic acid (**7**) (1.4‐fold decrease) and 4‐pentynoic acid (**8**) (∼3‐fold decrease), as well as a complete loss of activity with 4‐oxo‐pentanoic (**10**) acid (Table S6, Supporting Information). In contrast, conversion of 4,4,4‐trifluorobutyric acid (**9**) was further enhanced in the F430W/D449E background, yielding a 2.3‐fold improvement relative to D449E.

When the same acid panel was evaluated with SNAC (**3**) as the thiol, F430W/D449E showed increased overall conversion across most substrates compared to D449E, likely reflecting its enhanced utilization of **3**. The largest gains outside of propanoic (**4**) acid, which demonstrated a 3.4‐fold improvement, were observed for isobutyric (**6**) acid (1.5‐fold increase) and for 4,4,4‐trifluorobutyric (**9**) acid (2.9‐fold improvement). Notably, conversion of 4‐oxo‐pentanoic (**10**) acid was abolished in the F430W/D449E mutant, mirroring the loss of activity observed in the corresponding CoA‐dependent reactions. Despite efficient conversion of butyric acid to the corresponding acyl‐CoA by both enzymes, formation of butyryl‐SNAC (**5b**) was minimal, indicating that SNAC‐dependent activation imposes distinct steric or orientation constraints relative to CoA (**1**). A similar discrepancy was observed for 4‐pentynoic (**8**) acid. Across both thiol contexts, isobutyric acid (**6**) consistently emerged as the preferred substrate, with higher conversion than any other acid tested. Collectively, these results indicate that the F430W/D449E variant exhibits increased substrate discrimination relative to the D449E parent, with a pronounced preference for propanoic (**4**), isobutyric (**6**), and 4,4,4‐trifluorobutyric (**9**) acids, when SNAC is the thiol donor.

### 2.7 Functional Validation of the Engineered AcsA_*Pc*_ in a Polyketide Synthase System

Given the well‐established tolerance of polyketide synthase (PKS) domains for SNAC‐linked substrates, together with the ability of acyl‐CoA ligases to generate activated thioesters from short‐chain carboxylic acids, we hypothesized that the AcsA_*Pc*_ F430W/D449E variant could be used to generate acyl‐SNAC starter units that are able to supplement PKS loading and chain propagation. To test this concept, we employed a bimodular polyketide system, EryA1TE, comprising the first two modules of the 6‐deoxyerythronolide B synthase (DEBS) fuzed to the terminal DEBS thioesterase domain, and evaluated whether SNAC‐derived starter units, provided by AcsA_*Pc*_ F430W/D449E, could be loaded onto the PKS and propagated through subsequent extension and cyclization steps.

**FIGURE 6:**
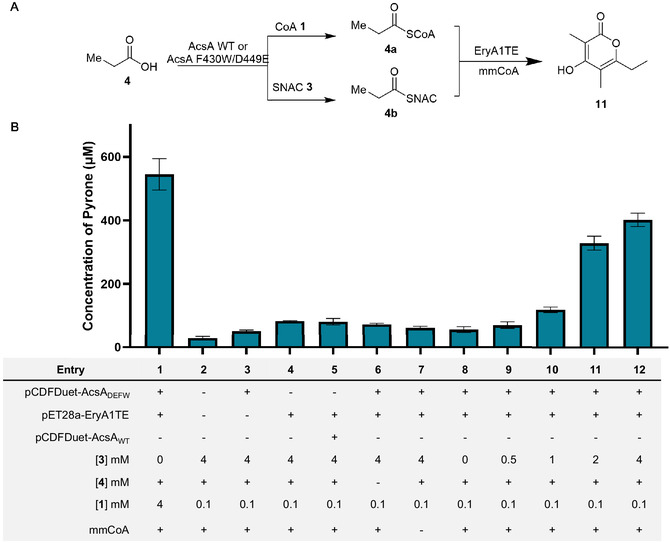
SNAC‐dependent pyrone formation in a reconstituted bimodular PKS system. Clarified *E. coli* lysates co‐expressing EryA1TE and AcsA*_Pc_* F430W/D449E were supplemented with propanoic acid, methylmalonyl‐CoA, and increasing concentrations of SNAC (**3**). (A) Scheme showing EryA1TE thiol competition assay. (B) Quantification of **11** production as a function of SNAC concentration. Product titers were determined relative to a triacetic lactone internal standard. Error bars represent the standard deviation of technical replicates (n = 3).

To validate the proof of concept and assess whether AcsA‐generated acyl‐SNACs could function as effective starter units for polyketide biosynthesis, we reconstituted the bimodular PKS system comprising EryA1TE in clarified *E. coli* lysates (Figure S6, Supporting Information), thereby enabling control of substrate concentrations and other assay components. EryA1TE and AcsA_*Pc*_ F430W/D449E were co‐expressed from separate plasmids in *E. coli* K207−3, and clarified lysates were used to enable precise control over methylmalonyl‐CoA and free **1** concentration. Reactions were conducted under conditions optimized for in vitro module activity and designed to approximate a physiological intracellular **1** concentration (250 µM) [19, 20, 21]. As a positive control, CoA (**1**) was first tested with the engineered AcsA variant. As expected, AcsA_*Pc*_ F430W/D449E efficiently converted propanoic (**4**) acid to propionyl‐CoA (**4a**) under these conditions, and the resulting starter unit was readily accepted by the PKS, resulting in robust pyrone (**11**) formation, as judged by LC‐MS analysis (entry 1, Figure 6B, Figure S7, Figure S8, Supporting Information, and Table S7, Supporting Information). Next, a series of reactions was developed in which SNAC (**3**) was supplied exogenously at concentrations ranging from 0 to 4 mM to compensate for the reduced efficiency with which PKS domains utilize SNAC‐linked starter units compared to native CoA thioesters. A series of control reactions was performed to validate pyrone formation in the EryA1TE system. A negative control derived from *E. coli* K207−3 that carried two “empty” plasmids was used to establish baseline pyrone production in the absence of PKS. Under these conditions, only trace amounts of a mass ion with the same *m*/*z* and retention time as the pyrone product were detected (entry 2, Figure 6B). Comparable low‐level signals were also observed in reactions that lacked EryA1TE or AcsA_*Pc*_ F430W/D449E (entries 3 and 4, respectively, Figure 6B), although at slightly elevated levels relative to when both enzymes were absent. Additionally, low levels of pyrone were detected when the AcsA_*Pc*_ F430W/D449E mutant was replaced with the wild‐type enzyme (entry 5, Figure 6B).

To confirm that the observed methyl pyrone (**11**) production arose specifically from the supplied starter unit, a negative control lacking **4** was evaluated. Under these conditions, pyrone formation remained at background levels comparable to those observed for other negative controls (entry 6, Figure 6B) and was markedly reduced relative to the positive control reaction containing **1** (entry 1). Together, these comparisons indicate that endogenous sources of **1** do not contribute appreciably to methyl pyrone formation in this system. Similarly, a control reaction lacking methylmalonyl‐CoA (mmCoA) showed less than 100 µM **11** production (entry 7, Figure 6B). This residual product formation likely reflects trace amounts of endogenous mmCoA present in *E. coli* K207−3, perhaps generated via exogenous propanoic acid and propionyl‐CoA ligase/carboxylase.

Finally, under optimized in vitro reaction conditions, the effect of increasing SNAC concentration (entries 8–12, Figure 6B) on **11** production by the EryA1TE system was systematically evaluated. A clear, dose‐dependent increase in **11** production was observed as **3** concentration increased. Quantification relative to a triacetic lactone (**12**) internal standard revealed that **11** titers increased from ∼56 µM in the absence of thiol **3** to ∼400 µM at 4 mM **3**, corresponding to ∼75% of the activity seen in the positive control in which CoA is the sole thiol source (entry 1). Notably, the absence of **3** led to only minimal pyrone accumulation, consistent with limited starter unit availability under these conditions.

These results indicate that AcsA_*Pc*_ F430W/D449E is active enough to provide a sufficient quantity of acyl‐SNAC starter units for a measurable increase in PKS‐mediated polyketide production. Moreover, the strong dependence of pyrone production on SNAC concentration demonstrates that engineered acyl‐CoA ligases can be used to modulate polyketide output by supplying orthogonal, non–CoA‐linked precursors in a controlled manner. Notably, pyrone formation remained low in the absence of exogenously supplied SNAC despite the presence of endogenous propionyl‐CoA derived from *E. coli* K207−3 metabolism, indicating that native starter unit pools are insufficient to support robust PKS initiation under these conditions and are likely further diluted upon cell lysis. In contrast, supplementation with SNAC and **1** provides a readily accessible and tunable source of activated starter units that bypasses these limitations. Importantly, identification of an AcsA variant with enhanced activity toward SNAC therefore offers a practical route to increasing polyketide titers through exogenous supplementation of inexpensive, membrane‐permeable precursors, while decoupling PKS output from fluctuations in native CoA‐linked metabolism.

## 3 Conclusions

Despite prior reports of SNAC activation by fatty acyl‐CoA ligases, existing systems are not well‐suited for generating short‐chain starter units for polyketide biosynthesis [22]. In contrast, the previously reported AcsA_*Pc*_ D449E variant exhibits broadened acid scope and detectable activity with synthetic thiols, making it a promising starting point for engineering SNAC‐dependent starter‐unit generation.

Using a combination of semi‐rational and random mutagenesis, AcsA_*Pc*_ libraries were subjected to high‐throughput colorimetric screening, yielding the F430W/D449E variant, which shows approximately 26‐fold improvement in propionyl‐SNAC formation relative to the wild‐type enzyme. Despite this substantial gain, overall conversion under endpoint conditions remains modest (13.2%), indicating room for further optimization. The use of (*R*)‐pantetheine as a surrogate substrate during screening likely limited the discovery of variants optimized specifically for SNAC, but this was necessary given the low baseline activity and signal‐to‐noise challenges associated with direct SNAC screening. Future efforts could focus on further assay optimization to more effectively resolve productive variants.

Using a combination of semi‐rational and random mutagenesis, high‐throughput screening of AcsAPc libraries identified the F430W/D449E variant, which exhibits enhanced activity with SNAC and a corresponding reduction in native CoA utilization. This shift in thiol preference represents an important step toward orthogonal starter‐unit generation, enabling enzymatic access to non‐CoA‐linked precursors while maintaining compatibility with downstream biosynthetic systems.

While SNAC thioesters have been widely employed as surrogate intermediates to bypass upstream PKS modules, fewer studies have explored enzymatic systems that generate acyl‐SNAC starter units in situ for direct incorporation into modular PKS assembly lines [15]. To our knowledge, this study represents the first demonstration of an in situ system in which enzymatically generated acyl‐SNACs serve to enhance downstream polyketide biosynthesis. Although fully in vivo validation was beyond the scope of this work, the reconstituted pyrone production system provides a clear demonstration that engineered acyl‐CoA ligases with enhanced SNAC activity can be leveraged to overcome the limiting starter unit pools exhibited by the in situ assay and boost polyketide output through controlled, exogenous supply of inexpensive starter‐unit precursors. Additionally, given the broad acid profile of AcsA_*Pc*_ F430W/D449E, this strategy offers a generalizable route for supplying diverse starter units to PKS assembly lines via short‐chain carboxylic acids supplied exogenously or via engineered metabolic pathways [23, 24]. Similar strategies may ultimately be extended to modulate extender‐unit supply, including methylmalonyl‐SNAC, in engineered biosynthetic pathways, potentially circumventing the need for membrane‐permeable malonic diesters or transport proteins [25, 26, 27].

Collectively, the F430W/D449E mutant and future iterations of AcsA_*Pc*_ provide access to a chemically diverse set of acyl‐SNACs directly relevant to polyketide biosynthesis. These results establish engineered acyl‐CoA ligases as versatile tools for overcoming precursor bottlenecks and expanding the accessible chemical space of polyketide starter units.

## 4 Materials and Methods

### 4.1 Overexpression and Purification of Wild‐Type and Variant AcsA_*Pc*_ Proteins

*Escherichia coli* BL21(DE3) chemically competent cells were transformed with pET28a‐AcsA_*Pc*_ plasmids encoding the desired variants (Figure S1 and Table S2, Supporting Information). Transformants were selected on LB agar plates supplemented with kanamycin (30 μg mL^−1^). Single colonies were used to inoculate LB medium (3 mL) containing kanamycin (30 μg mL^−1^) and grown at 37°C with shaking at 250 rpm for 18 h. Starter cultures were used to inoculate LB medium (300 mL) containing kanamycin (30 μg mL^−1^), which was incubated at 37°C with shaking at 250 rpm until an OD_600_ of ∼0.6 was reached. Protein expression was induced by the addition of IPTG to a final concentration of 1 mM, and cultures were incubated at 18°C for 18 h. Cells were harvested by centrifugation at 4816 g for 20 min, and the resulting pellets were resuspended in sodium phosphate buffer (20 mM, pH 7.4, 10 mL) containing NaCl (500 mM), imidazole (50 mM), and protease inhibitor (Pierce). Cells were lysed by sonication, and insoluble debris was removed by centrifugation at 15,000 g. The clarified lysate was passed through a 0.45 μm syringe filter and loaded onto a 1 mL HisTrap HP column using a Cytiva ÄKTAgo FPLC system. The column was washed with wash buffer (20 mM sodium phosphate, pH 7.4; 500 mM NaCl; 50 mM imidazole) until baseline absorbance was reached. Protein was eluted using elution buffer containing 20 mM sodium phosphate (pH 7.4), 500 mM NaCl, and 500 mM imidazole. Eluted fractions were concentrated using a Macrosep Advance 50 kDa MWCO centrifugal filter (Pall Corp., Puerto Rico) and stored in protein storage buffer (100 mM Tris‐HCl, 300 mM NaCl, pH 8.0, 10% v/v glycerol). Protein purity was assessed by SDS–PAGE densitometry, and concentrations were determined using the Bradford Protein Assay Kit (Bio‐Rad).

### 4.2 In Vitro Screening of AcsA Variants

A premixed reaction buffer consisting of sodium phosphate (200 mM, pH 7.0), MgCl_2_ (2 mM), (NH_4_)_2_SO_4_ (100 mM), ATP (0.4 mM), thiol of interest (0.2 mM), and propanoic acid (0.4 mM) was prepared and distributed into 150 μL aliquots. Reactions were initiated by the addition of purified AcsA_*Pc*_ variants to a final concentration of 0.05 μg μL^−1^ and incubated at room temperature for 1 h. Reactions were quenched by the addition of an equal volume of ice‐cold methanol and stored at −20°C overnight prior to analysis.

### 4.3 Ellman's Assay

Ellman's assays were performed as previously described with minor modifications. Individual colonies of *E. coli* BL21(DE3) expressing AcsA_*Pc*_ D449E variants were used to inoculate LB medium (1 mL) containing kanamycin (30 μg mL^−1^) in round‐bottom 96‐deep‐well plates (VWR). Plates were sealed with AeraSeal breathable film and incubated at 37°C with shaking at 300 rpm for 18 h. Aliquots (50 μL) were transferred to fresh deep‐well plates containing LB medium (1 mL) and kanamycin (30 μg mL^−1^), and the plates were incubated for 3 h under identical conditions. Protein expression was induced by adding IPTG (1 mM final concentration), and cultures were incubated at 15°C with shaking at 300 rpm for 18 h. Cells were harvested by centrifugation at 4816 g for 15 min and resuspended in Tris‐HCl buffer (100 mM, pH 8.0) containing lysozyme (5 mg mL^−1^). Following a 20 min incubation at room temperature, cells were subjected to a single freeze–thaw cycle. Cell debris was removed by centrifugation at 4816 g for 15 min. Clarified lysate (50 μL) was combined with reaction buffer (200 μL) containing sodium phosphate (200 mM, pH 7.0), MgCl_2_ (2 mM), (NH_4_)_2_SO_4_ (100 mM), ATP (0.5 mM), thiol of interest (0.2 mM), and propanoic acid (0.5 mM) in a transparent 96‐well plate (Greiner Bio‐One). Reactions were incubated at room temperature for 18 h.

Ellman's reagent (13 μL), prepared by dissolving 154.2 mg of 5,5′‐dithiobis(2‐nitrobenzoic acid) in 50 mL of 0.1 M sodium phosphate buffer (pH 7.0), was added to each well. Plates were incubated for 10 min at room temperature, and absorbance at 412 nm was measured using a Synergy H1 microplate reader (BioTek). Secondary screening was performed by inoculating three replicate wells per hit variant and repeating the assay to confirm activity.

### 4.4 Preparation of *E. coli* Lysates Containing EryA1TE and AcsA

*E. coli* BL21(DE3) cells were transformed with pET28a‐EryA1TE (Figure S1 and Tables S2–S3, Supporting Information) and selected on LB agar containing kanamycin (30 μg mL^−1^). Single colonies were used to inoculate LB medium (3 mL) with kanamycin (30 μg mL^−1^) and grown at 37°C with shaking at 250 rpm for 18 h. Cultures were expanded into LB medium (20 mL) and grown to an OD_600_ of ∼0.4. Cells were harvested by centrifugation at 4816 g for 7 min, washed three times with 0.1 M CaCl_2_, and resuspended in 1 mL of 0.1 M CaCl_2_ and 1 mL of 20% (v/v) glycerol. These competent cells were transformed with pCDFDuet‐ AcsA_*Pc*_. Transformants were selected on LB agar containing kanamycin (20 μg mL^−1^) and streptomycin (20 μg mL^−1^). Starter cultures were grown in LB medium (3 mL) containing both antibiotics for 18 h and used to inoculate Terrific Broth (300 mL) supplemented with kanamycin (20 μg mL^−1^) and streptomycin (20 μg mL^−1^). Protein expression was induced with IPTG (1 mM), and cultures were incubated at 18°C for 24 h. Cells were harvested by centrifugation at 4816 g for 20 min and resuspended in 100 mM NaH_2_PO_4_ buffer (pH 7.2) containing EDTA (1 mM), glycerol (20% v/v), and protease inhibitor (Pierce). Cells were lysed by sonication, and clarified lysates were obtained by centrifugation at 10,000 g for 1 h, filtered through a 0.45 μm syringe filter, and stored at −80°C until use.

### 4.5 In Situ Pyrone Formation Using Acyl‐SNACs

Reaction mixtures containing phosphate buffer (100 mM, pH 7.0), propionic acid (4 mM), methylmalonyl‐CoA (3.5 mM), and varying concentrations of CoA (**1**) or SNAC (**3**) were prepared and distributed into 50 μL aliquots. Clarified lysate containing AcsA_*Pc*_ F430W/D449E and EryA1TE was added to initiate the reaction. Reactions were incubated at room temperature for 47 h, quenched with ice‐cold methanol, and stored at −20°C prior to LC–MS analysis.

## Supporting Information

Additional supporting information can be found online in the supporting information section.

## Author Contributions

J. R. C. and G. J. W. conceived the project. S.A.T. designed and performed the AcsA library construction, screening, other mutagenesis, and enzyme assays, with supervision from J. R. C. J. R. C. designed and performed the AcsA acid/thiol specificity studies and EryA1TE experiments. All authors analyzed the data. G. J. W. supervised the research. J. R. C. and G. J. W. wrote the manuscript with input from S.A.T.

## Funding

This work was supported by the National Institutes of General Medical Sciences (GM124112).

## Conflicts of Interest

The authors declare no conflicts of interest.

## Supporting information

Supplementary Material

## Data Availability

The data that support the findings of this study are available in the supplementary material of this article. Additional raw data are available from the corresponding author upon reasonable request.

## References

[1] E. R. Marella , C. Holkenbrink , V. Siewers , and I. Borodina , “Engineering Microbial Fatty Acid Metabolism for Biofuels and Biochemicals,” Current Opinion in Biotechnology 50 (2018): 39–46. 29101852 10.1016/j.copbio.2017.10.002

[2] A. M. Gulick , “Conformational Dynamics in the Acyl‐CoA Synthetases, Adenylation Domains of Non‐Ribosomal Peptide Synthetases, and Firefly Luciferase,” ACS Chemical Biology 4, no. 10 (2009): 811–827. 19610673 10.1021/cb900156h PMC2769252

[3] A. M. Soohoo , D. P. Cogan , K. L. Brodsky , and C. Khosla , “Structure and Mechanisms of Assembly‐Line Polyketide Synthases,” Annual Review of Biochemistry 93, no. 1 (2024): 471–498. 10.1146/annurev-biochem-080923-043654 PMC11907408 38663033

[4] Y. ‐N. Choi , J. W. Lee , J. W. Kim , and J. M. Park , “Acetyl‐CoA‐Derived Biofuel and Biochemical Production in Cyanobacteria: A Mini Review,” Journal of Applied Phycology 32, no. 3 (2020): 1643–1653.

[5] L. M. Fulton , L. R. Lynd , A. Körner , N. Greene , and L. R. Tonachel , “The Need for Biofuels as Part of a Low Carbon Energy Future,” Biofuels, Bioproducts and Biorefining 9, no. 5 (2015): 476–483.

[6] B. C. H. Moore , “Biosynthesis and Attachment of Novel Bacterial Polyketide Synthase Starter Units,” Natural Product Reports 19, no. 1 (2002): 70–99. 11902441 10.1039/b003939j

[7] Z. L. Fowler , W. W. Gikandi , and M. A. G. Koffas , “Increased Malonyl Coenzyme A Biosynthesis by Tuning the, *Escherichia Coli*, Metabolic Network and Its Application to Flavanone Production,” Applied and Environmental Microbiology 75, no. 18 (2009): 5831–5839. 19633125 10.1128/AEM.00270-09 PMC2747866

[8] R. van der Sluis and E. Erasmus , “Xenobiotic/Medium Chain Fatty Acid: CoA Ligase – a Critical Review on Its Role in Fatty Acid Metabolism and the Detoxification of Benzoic Acid and Aspirin,” Expert Opinion on Drug Metabolism & Toxicology 12, no. 10 (2016): 1169–1179. 27351777 10.1080/17425255.2016.1206888

[9] L. M. M. Mouterde , G. Willig , M. M. J. Langlait , F. Brunois , M. Chadni , and F. Allais , “Unlocking the Access to Oxidized Coenzyme A via a Single‐Step Green Membrane‐Based Purification,” Scientific Reports 12, no. 1 (2022): 12991. 35906370 10.1038/s41598-022-17250-8 PMC9338019

[10] L. M. M. Mouterde and J. D. Stewart , “Isolation and Synthesis of One of the Most Central Cofactors in Metabolism: Coenzyme A,” Organic Process Research & Development 23, no. 1 (2019): 19–30.

[11] S. Klopries , U. Sundermann , and F. Schulz , “Quantification of *N* ‐Acetylcysteamine Activated Methylmalonate Incorporation into Polyketide Biosynthesis,” Beilstein Journal of Organic Chemistry 9 (2013): 664–674. 23616811 10.3762/bjoc.9.75 PMC3628877

[12] T. Q. Paulsel and G. J. Williams , “Current State‐of‐the‐Art Toward Chemoenzymatic Synthesis of Polyketide Natural Products,” Chembiochem : A European Journal of Chemical Biology 24, no. 21 (2023): e202300386. 37615926 10.1002/cbic.202300386 PMC10964317

[13] J. Franke and C. Hertweck , “Biomimetic Thioesters as Probes for Enzymatic Assembly Lines: Synthesis, Applications, and Challenges,” Cell Chemical Biology 23, no. 10 (2016): 1179–1192. 27693058 10.1016/j.chembiol.2016.08.014

[14] A. J. Hughes and A. Keatinge‐Clay , “Enzymatic Extender Unit Generation for In Vitro Polyketide Synthase Reactions: Structural and Func‐Tional Showcasing of Streptomyces Coelicolor MatB,” Chemistry & Biology 18, no. 2 (2011): 165–176. 21338915 10.1016/j.chembiol.2010.12.014

[15] J. R. Cossin , T. Q. Paulsel , K. Castelli , B. Wcisel , A. A. Malico , and G. J. Williams , “Engineering the Specificity of Acetyl‐CoA Synthetase for Diverse Acyl‐CoA Thioester Generation,” ACS Chemical Biology 20, no. 4 (2025): 930–941. 40176419 10.1021/acschembio.5c00014 PMC12483075

[16] J. Jumper , R. Evans , A. Pritzel , et al., “Highly Accurate Protein Structure Prediction with AlphaFold,” Nature 596, no. 7873 (2021): 583–589. 34265844 10.1038/s41586-021-03819-2 PMC8371605

[17] G. L. Ellman , “Tissue Sulfhydryl Groups,” Archives of Biochemistry and Biophysics 82, no. 1 (1959): 70–77. 13650640 10.1016/0003-9861(59)90090-6

[18] P. W. Riddles , R. L. Blakeley , and B. Zerner , “Ellman's Reagent: 5,5′‐Dithiobis(2‐Nitrobenzoic Acid)—a Reexamination,” Analytical Biochemistry 94, no. 1 (1979): 75–81. 37780 10.1016/0003-2697(79)90792-9

[19] R. Vadali , “Cofactor Engineering of Intracellular CoA/Acetyl‐CoA and Its Effect on Metabolic Flux Redistribution in Escherichia Coli,” Metabolic Engineering 6, no. 2 (2004): 133–139. 15113566 10.1016/j.ymben.2004.02.001

[20] D. Radoš , S. Donati , M. Lempp , J. Rapp , and H. Link , “Homeostasis of the Biosynthetic E. Coli Metabolome,” iScience 25, no. 7 (2022): 104503. 35754712 10.1016/j.isci.2022.104503 PMC9218372

[21] J. Kim and K. H. Kim , “Effects of Minimal Media vs. Complex Media on the Metabolite Profiles of Escherichia Coli and Saccharomyces Cerevisiae,” Process Biochemistry 57 (2017): 64–71.

[22] P. Arora , A. Vats , P. Saxena , D. Mohanty , and R. S. Gokhale , “Promiscuous Fatty Acyl CoA Ligases Produce Acyl‐CoA and Acyl‐SNAC Precursors for Polyketide Biosynthesis,” Journal of the American Chemical Society 127, no. 26 (2005): 9388–9389. 15984864 10.1021/ja052991s

[23] F. Xu , M. Hong , M. Z. Huang , et al., “Exploring the Metabolic Fate of Propanol in Industrial Erythromycin‐Producing Strain via 13C Labeling Experiments and Enhancement of Erythromycin Production by Rational Metabolic Engineering of Saccharopolyspora Erythraea,” Biochemical and Biophysical Research Communications 542 (2021): 73–79. 33497965 10.1016/j.bbrc.2021.01.024

[24] Z. Xu , M. Wang , and B. ‐C. Ye , “TetR Family Transcriptional Regulator PccD Negatively Controls Propionyl Coenzyme A Assimilation in Saccharopolyspora Erythraea,” Journal of Bacteriology 199, no. 20 (2017): e00281–e00217. 28760847 10.1128/JB.00281-17 PMC5637179

[25] Y. A. Chan , A. M. Podevels , B. M. Kevany , and M. G. Thomas , “Biosynthesis of Polyketide Synthase Extender Units,” Natural Product Reports 26, no. 1 (2009): 90–114. 19374124 10.1039/b801658p PMC2766543

[26] S. H. Klass , M. Wesselkamper , A. E. Cowan , et al., “Engineering Controllable Alteration of Malonyl‐CoA Levels to Enhance Polyketide Production,” Nature Chemical Biology 21, no. 8 (2025): 1214–1225. 40500421 10.1038/s41589-025-01911-6 PMC12303837

[27] J. H. An and Y. S. Kim , “A Gene Cluster Encoding Malonyl‐CoA Decarboxylase (MatA), Malonyl‐CoA Synthetase (MatB) and a Putative Dicarboxylate Carrier Protein (MatC) in *Rhizobium Trifolii* ,” European Journal of Biochemistry 257, no. 2 (1998): 395–402. 9826185 10.1046/j.1432-1327.1998.2570395.x

