# Supplementary material for: Engineering an Acyl‐CoA Ligase With Enhanced Activity Toward Synthetic CoA Alternatives
Source: Chembiochem. 2026 May 13;27(10):e70385. doi: 10.1002/cbic.70385 (PMC13168922; doi:10.1002/cbic.70385)
Supplement: Supplementary file 1 — Supplementary Material [file CBIC-27-e70385-s001.pdf]

Engineering an Acyl-CoA Ligase with Enhanced Activity Toward Synthetic CoA Alternatives

Jared R. Cossin,[1+] Sarah A. Taboada,[1,3+] Gavin J. Williams*[1,2]

[1] North Carolina State University, Raleigh, North Carolina, 27695, United States
[2] Comparative Medicine Institute, Raleigh, North Carolina, 27695, United States
[3] Present address: Florida State University, Tallahassee, Fl 32306-4390, United States
[+] Co-first authors
* Corresponding author: gjwillia@ncsu.edu

Table of Contents

Supplemental Tables

Supplementary Table S1. Low-resolution LCMS and HPLC analysis of initial alternative thiol activity with WT AcsA and AcsA D449E.

Supplementary Table S2. Sequences of DNA Constructs.

Supplementary Table S3. *E. coli* strains used in this study.

Supplementary Table S4. Oligonucleotides used in this study.

Supplementary Table S5. HPLC and LCMS analysis of AcsA double and triple mutants with alternative thiols.

Supplementary Table S6. HPLC and LCMS analysis of select AcsA mutants with various acids.

Supplementary Table S7. LCMS analysis of *in situ* pyrone production assays.

Supplemental Figures

Supplementary Figure S1. Plasmid map of pET28a-AcsA.

Supplementary Figure S2. SDS page gel of purified AcsA mutants.

Supplementary Figure S3. Time course assays for various AcsA mutants with different thiols

Supplementary Figure S4. Representative LCMS chromatograms observed in the AcsA acid panel testing.

Supplementary Figure S5. Plasmid maps of pET28a-EryA1TE and pCDFduet-AcsA.

Supplementary Figure S6. SDS page gel of purified EryA1TE and $AcsA_{Pc}$.

Supplementary Figure S7. LCMS chromatograms demonstrating *in situ* pyrone formation.

Supplementary Figure S8. Scheme showing a mechanism for pyrone formation via EryA1TE

Supplemental Methods

HPLC Analysis of quenched AcsA Catalyzed Reactions.

LCMS Analysis of quenched AcsA Catalyzed Reactions.

Model Generation of $AcsA_{\mathit{Pc.}}$

Docking Study $AcsA_{\mathit{Pc.}}$

Saturation and Random Mutagenesis of AcsA.

Synthesis of HSNAC.

Testing of Initial Reaction Rates.

Acid Panel Testing.

Supplemental Tables

Supplementary Table S1. Low-resolution LCMS and HPLC analysis of initial alternative thiol activity with WT AcsA and AcsA D449E.

		Thiol Substrate					Product					
Construct	Thiol/ Product	Retention Time (min)	Calculated Mass $[M+H]^+$	Observed Mass $[M+H]^+$	Δ Mass (Calc. – Obs.)	Peak Area	Retention Time (min)	Calculated Mass $[M+H]^+$	Observed Mass $[M+H]^+$	Δ Mass (Calc. – Obs.)	Peak Area	% Conversion[c]
WT AcsA	1/2 [a]	15.3	768.1	768.0	0.1	29,000	24.1	ND	ND	ND	608,000	95.3 ± 2.3
	3/4 [b]	3.63	279.4	279.4	0	3,649,000	4.05	335.1	335.1	0	79,000	2.2 ± 0.1
	5/6 [b]	3.41	120.1	120.0	0.1	953,000	4.15	176.1	176.2	0.1	2,900	0.3 ± 0.1
D449E	1/2 [a]	15.3	768.1	768.0	0.1	8,200	24.1	ND	ND	ND	694,000	97.7 ± 1.2
	3/4 [b]	3.63	279.4	279.4	0	3,233,000	4.05	335.1	335.1	0	520,000	12.8 ± 1.3
	5/6 [b]	3.41	120.1	120.0	0.1	929,000	4.15	176.1	176.2	0.1	43,000	3.9 ± 0.7

[a] Reactions that utilized CoA as the thiol of interest were analyzed via HPLC using methods described in the Supplemental Methods section

[b] Reactions that utilized SNAC or Pantetheine as the thiol of interest were analyzed via LCMS according to the method described in the Supplemental Methods.

[c] Percent conversion was calculated by dividing the peak area of the acyl-thiol product by the combined peak areas of the acyl-thiol product and unreacted thiol.

Supplementary Table S2. Sequences of DNA constructs. AcsA$_{Pc}$ (GenBank Accession #AB125061) is highlighted in green. EryA1TE (GenBank Accession #AM420293.1) is bolded.

Construct Name	DNA Sequence
pET28a-AcsA$_{Pc}$	TGGCGAATGGGACGCGCCCTGTAGCGGCGCATTAAGCGCGGCGGGTG<br>TGGTGGTTACGCGCAGCGTGACCGCTACACTTGCCAGCGCCCTAGCG<br>CCCGCTCCTTTCGCTTTCTTCCCTTCCTTTCTCGCCACGTTCGCCGGCT<br>TTCCCCGTCAAGCTCTAAATCGGGGGCTCCCTTTAGGGTTCCGATTTAG<br>TGCTTTACGGCACCTCGACCCCAAAAAACTTGATTAGGGTGATGGTTCA<br>CGTAGTGGGCCATCGCCCTGATAGACGGTTTTTCGCCCTTTGACGTTG<br>GAGTCCACGTTCTTTAATAGTGGACTCTTGTTCCAAACTGGAACAACAC<br>TCAACCCTATCTCGGTCTATTCTTTTGATTTATAAGGGATTTTGCCGATTT<br>CGGCCTATTGGTTAAAAAATGAGCTGATTTAACAAAAATTTAACGCGAAT<br>TTTAACAAAATATTAACGTTTACAATTTCAGGTGGCACTTTTCGGGGAAA<br>TGTGCGCGGAACCCCTATTTGTTTATTTTTCTAAATACATTCAAATATGTAT<br>CCGCTCATGAATTAATTCTTAGAAAAACTCATCGAGCATCAAATGAAACT<br>GCAATTTATTCATATCAGGATTATCAATACCATATTTTTGAAAAAGCCGTTT<br>CTGTAATGAAGGAGAAAACTCACCGAGGCAGTTCCATAGGATGGCAAG<br>ATCCTGGTATCGGTCTGCGATTCCGACTCGTCCAACATCAATACAACCT<br>ATTAATTTCCCCTCGTCAAAAATAAGGTTATCAAGTGAGAAATCACCATG<br>AGTGACGACTGAATCCGGTGAGAATGGCAAAAGTTTATGCATTTCTTTC<br>CAGACTTGTTCAACAGGCCAGCCATTACGCTCGTCATCAAAATCACTCG<br>CATCAACCAAACCGTTATTCATTCGTGATTGCGCCTGAGCGAGACGAAA<br>TACGCGATCGCTGTTAAAAGGACAATTACAAACAGGAATCGAATGCAAC<br>CGGCGCAGGAACACTGCCAGCGCATCAACAATATTTTCACCTGAATCAG<br>GATATTCTTCTAATACCTGGAATGCTGTTTTCCCGGGGATCGCAGTGGT<br>GAGTAACCATGCATCATCAGGAGTACGGATAAAATGCTTGATGGTCGGA<br>AGAGGCATAAATTCCGTCAGCCAGTTTAGTCTGACCATCTCATCTGTAAC<br>ATCATTGGCAACGCTACCTTTGCCATGTTTCAGAAACAACTCTGGCGCA<br>TCGGGCTTCCCATACAATCGATAGATTGTCGCACCTGATTGCCCGACAT<br>TATCGCGAGCCCATTTATACCCATATAAATCAGCATCCATGTTGGAATTTA<br>ATCGCGGCCTAGAGCAAGACGTTTCCCGTTGAATATGGCTCATAACACC<br>CCTTGTATTACTGTTTATGTAAGCAGACAGTTTTATTGTTCATGACCAAAA<br>TCCCTTAACGTGAGTTTTCGTTCCACTGAGCGTCAGACCCCGTAGAAAA<br>GATCAAAGGATCTTCTTGAGATCCTTTTTTTCTGCGCGTAATCTGCTGCT<br>TGCAAACAAAAAAACCACCGCTACCAGCGGTGGTTTGTTTGCCGGATC<br>AAGAGCTACCAACTCTTTTTCCGAAGGTAACTGGCTTCAGCAGAGCGC<br>AGATACCAAATACTGTCCTTCTAGTGTAGCCGTAGTTAGGCCACCACTTC<br>AAGAACTCTGTAGCACCGCCTACATACCTCGCTCTGCTAATCCTGTTAC<br>CAGTGGCTGCTGCCAGTGGCGATAAGTCGTGTCTTACCGGGTTGGACT<br>CAAGACGATAGTTACCGGATAAGGCGCAGCGGTCGGGCTGAACGGGG<br>GGTTCGTGCACACAGCCCAGCTTGGAGCGAACGACCTACACCGAACT<br>GAGATACCTACAGCGTGAGCTATGAGAAAGCGCCACGCTTCCCGAAGG<br>GAGAAAGGCGGACAGGTATCCGGTAAGCGGCAGGGTCGGAACAGGAG<br>AGCGCACGAGGGAGCTTCCAGGGGGAAACGCCTGGTATCTTTATAGTC<br>CTGTCGGGTTTCGCCACCTCTGACTTGAGCGTCGATTTTTGTGATGCTC<br>GTCAGGGGGGCGGAGCCTATGGAAAAACGCCAGCAACGCGGCCTTTT<br>TACGGTTCCTGGCCTTTTGCTGGCCTTTTGCTCACATGTTCTTTCCTGC<br>GTTATCCCCTGATTCTGTGGATAACCGTATTACCGCCTTTGAGTGAGCT<br>GATACCGCTCGCCGCAGCCGAACGACCGAGCGCAGCGAGTCAGTGAG

	CGAGGAAGCGGAAGAGCGCCTGATGCGGTATTTTCTCCTTACGCATCT<br>GTGCGGTATTTCACACCGCATATATGGTGCACTCTCAGTACAATCTGCTC<br>TGATGCCGCATAGTTAAGCCAGTATACACTCCGCTATCGCTACGTGACT<br>GGGTCATGGCTGCGCCCCGACACCCGCCAACACCCGCTGACGCGCCC<br>TGACGGGCTTGTCTGCTCCCGGCATCCGCTTACAGACAAGCTGTGACC<br>GTCTCCGGGAGCTGCATGTGTCAGAGGTTTTCACCGTCATCACCGAAA<br>CGCGCGAGGCAGCTGCGGTAAAGCTCATCAGCGTGGTCGTGAAGCGA<br>TTCACAGATGTCTGCCTGTTCATCCGCGTCCAGCTCGTTGAGTTTCTCC<br>AGAAGCGTTAATGTCTGGCTTCTGATAAAGCGGGCCATGTTAAGGGCG<br>GTTTTTTCCTGTTTGGTCACTGATGCCTCCGTGTAAGGGGGATTTCTGT<br>TCATGGGGGTAATGATACCGATGAAACGAGAGAGGATGCTCACGATACG<br>GGTTACTGATGATGAACATGCCCGGTTACTGGAACGTTGTGAGGGTAAA<br>CAACTGGCGGTATGGATGCGGCGGGACCAGAGAAAAATCACTCAGGGT<br>CAATGCCAGCGCTTCGTTAATACAGATGTAGGTGTTCCACAGGGTAGCC<br>AGCAGCATCCTGCGATGCAGATCCGGAACATAATGGTGCAGGGCGCTG<br>ACTTCCGCGTTTCCAGACTTTACGAAACACGGAAACCGAAGACCATTCA<br>TGTTGTTGCTCAGGTCGCAGACGTTTTGCAGCAGCAGTCGCTTCACGT<br>TCGCTCGCGTATCGGTGATTCATTCTGCTAACCAGTAAGGCAACCCCGC<br>CAGCCTAGCCGGGTCCTCAACGACAGGAGCACGATCATGCGCACCCG<br>TGGGGCCGCCATGCCGGCGATAATGGCCTGCTTCTCGCCGAAACGTTT<br>GGTGGCGGGACCAGTGACGAAGGCTTGAGCGAGGGCGTGCAAGATTC<br>CGAATACCGCAAGCGACAGGCCGATCATCGTCGCGCTCCAGCGAAAG<br>CGGTCCTCGCCGAAAATGACCCAGAGCGCTGCCGGCACCTGTCCTAC<br>GAGTTGCATGATAAAGAAGACAGTCATAAGTGCGGCGACGATAGTCATG<br>CCCCGCGCCCACCGGAAGGAGCTGACTGGGTTGAAGGCTCTCAAGGG<br>CATCGGTCGAGATCCCGGTGCCTAATGAGTGAGCTAACTTACATTAATT<br>GCGTTGCGCTCACTGCCCGCTTTCCAGTCGGGAAACCTGTCGTGCCA<br>GCTGCATTAATGAATCGGCCAACGCGCGGGGAGAGGCGGTTTGCGTAT<br>TGGGCGCCAGGGTGGTTTTTCTTTTCACCAGTGAGACGGGCAACAGCT<br>GATTGCCCTTCACCGCCTGGCCCTGAGAGAGTTGCAGCAAGCGGTCC<br>ACGCTGGTTTGCCCCAGCAGGCGAAAATCCTGTTTGATGGTGGTTAAC<br>GGCGGGATATAACATGAGCTGTCTTCGGTATCGTCGTATCCCACTACCG<br>AGATATCCGCACCAACGCGCAGCCCGGACTCGGTAATGGCGCGCATTG<br>CGCCCAGCGCCATCTGATCGTTGGCAACCAGCATCGCAGTGGGAACG<br>ATGCCCTCATTCAGCATTTGCATGGTTTGTTGAAAACCGGACATGGCAC<br>TCCAGTCGCCTTCCCGTTCCGCTATCGGCTGAATTTGATTGCGAGTGAG<br>ATATTTATGCCAGCCAGCCAGACGCAGACGCGCCGAGACAGAACTTAAT<br>GGGCCCGCTAACAGCGCGATTTGCTGGTGACCCAATGCGACCAGATGC<br>TCCACGCCCAGTCGCGTACCGTCTTCATGGGAGAAAATAATACTGTTGA<br>TGGGTGTCTGGTCAGAGACATCAAGAAATAACGCCGGAACATTAGTGC<br>AGGCAGCTTCCACAGCAATGGCATCCTGGTCATCCAGCGGATAGTTAAT<br>GATCAGCCCACTGACGCGTTGCGCGAGAAGATTGTGCACCGCCGCTTT<br>ACAGGCTTCGACGCCGCTTCGTTCTACCATCGACACCACCACGCTGGC<br>ACCCAGTTGATCGGCGCGAGATTTAATCGCCGCGACAATTTGCGACGG<br>CGCGTGCAGGGCCAGACTGGAGGTGGCAACGCCAATCAGCAACGACT<br>GTTTGCCCGCCAGTTGTTGTGCCACGCGGTTGGGAATGTAATTCAGCT<br>CCGCCATCGCCGCTTCCACTTTTTCCCGCGTTTTCGCAGAAACGTGGC<br>TGGCCTGGTTCACCACGCGGGAAACGGTCTGATAAGAGACACCGGCAT<br>ACTCTGCGACATCGTATAACGTTACTGGTTTCACATTCACCACCCTGAAT<br>TGACTCTCTTCCGGGCGCTATCATGCCATACCGCGAAAGGTTTTGCGC<br>CATTCGATGGTGTCCGGGATCTCGACGCTCTCCCTTATGCGACTCCTG

	CATTAGGAAGCAGCCCAGTAGTAGGTTGAGGCCGTTGAGCACCGCCGC<br>CGCAAGGAATGGTGCATGCAAGGAGATGGCGCCCAACAGTCCCCCGG<br>CCACGGGGCCTGCCACCATACCCACGCCGAAACAAGCGCTCATGAGC<br>CCGAAGTGGCGAGCCCGATCTTCCCCATCGGTGATGTCGGCGATATAG<br>GCGCCAGCAACCGCACCTGTGGCGCCGGTGATGCCGGCCACGATGC<br>GTCCGGCGTAGAGGATCGAGATCTCGATCCCGCGAAATTAATACGACTC<br>ACTATAGGGGAATTGTGAGCGGATAACAATTCCCCTCTAGAAATAATTTT<br>GTTTAACTTTAAGAAGGAGATATACCATGGGCAGCAGCCATCATCATCAT<br>CATCACAGCAGCGGCCTGGTGCCGCGCGGCAGCCATATGGCTAGCAT<br>GACTGGTGGACAGCAAATGGGTCGCGGATCCGAATTCATGCGGGACTA<br>TGAGCACGTGGTGGAGAGCTTCGACTATCTGCAGAGCGCGACCCAGG<br>ACCTGCACGGCGAGCTGACCGCCCTGAACGCCTGCGTGGAGTGCTGC<br>GACCGTCACGCCCACGGCGAGGCCGTCGCGCTGTACTGCGAGGCCC<br>AGGACGGCCACGCGGAGCGCTACCGCTTCCGCGACCTGCAGCGCCA<br>GGCCGCGCGCTTCGGCAACTTCCTGCGCGAGCAGGGCGTGAAGCCG<br>GGTGACCGTGTGGCCGGCCTGATGCCGCGCACCGTGGAGCTGCTGAT<br>CGCCATCCTGGGTACCTGGCGTATCGGTGCCGTCTACCAGCCGCTGTT<br>CACCGCCTTCGGTCCGAAGGCCATCGAGCAGCGCCTGAACTGCAGCA<br>ACGCCCGTTGGATCGTGACCGACCCGCACAACCGTCCGAAGCTGGAC<br>GACGTCACCGACTGCCCGAGCATCGTGGTCACCGGCGGCGCCCCGCA<br>GAACCCGGCCGACCACCACTTCTGGAGCGCCCTGAACCGCCAGGCCG<br>ACGACTGCGCCCCGGTGCTGCTGGACGCCAGCGCCCCGTTCCTGCTG<br>ATGTGCACCAGCGGTACCACCGGTCCGGCCAAGCCGCTGGAAGTGCC<br>GCTGAGCGCCATCCTGGCGTTCAAGGGCTACATGCGTGACGCCATCGA<br>CCTGCGCGCCGACGACCGTTTCTGGAACCTGGCGGACCCGGGTTGG<br>GCGTACGGCCTGTACTACGCGGTGACCGGTCCGCTGGCCTGCGGTTA<br>CGCCACCCTGTTCTACGACGGTCCGTTCACCGTGGAGAGCACCCGCC<br>ACATCATCGCCAAGTACGCGATCAACAACCTGGCGGGTAGCCCGACCG<br>CCTACCGCTTCCTGATCGCCGCGGGCGCGGAGTTCGCGGACGCCGTG<br>CGTGGCCGCCTGCGTGCCGTCAGCAGCGCGGGCGAGCCGCTGAACC<br>CGCAGGTGGTCCGTTGGTTCGCGGAGCAGCTGGGCGTGGTCATCCAC<br>GACCACTACGGCCAGACCGAGATCGGCATGGTGCTGTGCAACCACCA<br>CGGCCTGCGTCACCCGGTGCGTGAGGGTAGCGCCGGCTACGCCGTC<br>CCGGGTTACCGTATCGTGGTCCTGGACAAAGCCCACCGTGAGCTGCC<br>GGCCGGTCAGCCGGGCGTGCTGGCCGTCGACCGCGAGCGCAGCCCG<br>CTGTGCTGGTTCGACGGTTACCTGGGTATGCCGACCCAGGCCTTCGCC<br>GGCCGCTACTACCTGAGCGGCGACATCGTGGAGCTGAACGACGACGG<br>CAGCATCAGCTTCGTCGGCCGCAACGACGACCTGATCACCACCAGCG<br>GTTACCGCGTGGGTCCGTTCGACGTGGAGAGCGCCCTGATCGAGCAC<br>CCGGCCGTGGTGGAGGCGGCCGTCATCGGCAAGCCGGACCCGCAGC<br>GCACCGAGCTGATCAAGGCCTTCGTGGTCCTGAACACCCCGTACCTGC<br>CGAGCCCGGAGCTGGCGGAGGAGCTGCGTCTGCACGTGCGTCAGCG<br>TCTGGCCGCGCACGCCTACCCGCGCGAGATGGAGTTCGTCGACCACC<br>TGCCGAAGACCCCGAGCGGCAAACTGCAGCGGTTCATCCTGCGTAAC<br>CAGGAGATCGCCAAACAGCAGGCCCTGGGCTAAAAGCTTGCGGCCGC<br>ACTCGAGCACCACCACCACCACCACTGAGATCCGGCTGCTAACAAAGC<br>CCGAAAGGAAGCTGAGTTGGCTGCTGCCACCGCTGAGCAATAACTAGC<br>ATAACCCCTTGGGGCCTCTAAACGGGTCTTGAGGGGTTTTTTGCTGAAA<br>GGAGGAACTATATCCGGAT
pET28a-<br>EryA1TE	CCACCGCTGAGCAATAACTAGCATAACCCCTTGGGGCCTCTAAACGGGTCTTG<br>AGGGGTTTTTTGCTGAAAGGAGGAACTATATCCGGATTGGCGAATGGGACGCG

	CCCTGTAGCGGCGCATTAAGCGCGGCGGGTGTGGTGGTTACGCGCAGCGTGA<br>CCGCTACACTTGCCAGCGCCCTAGCGCCCGCTCCTTTCGCTTTCTTCCCTTCC<br>TTTCTCGCCACGTTCGCCGGCTTTCCCCGTCAAGCTCTAAATCGGGGGCTCCC<br>TTTAGGGTTCCGATTTAGTGCTTTACGGCACCTCGACCCCAAAAAACTTGATTA<br>GGGTGATGGTTCACGTAGTGGGCCATCGCCCTGATAGACGGTTTTTCGCCCTT<br>TGACGTTGGAGTCCACGTTCTTTAATAGTGGACTCTTGTTCCAAACTGGAACAA<br>CACTCAACCCTATCTCGGTCTATTCTTTTGATTTATAAGGGATTTTGCCGATTTCG<br>GCCTATTGGTTAAAAAATGAGCTGATTTAACAAAAATTTAACGCGAATTTTAACAA<br>AATATTAACGTTTACAATTTCAGGTGGCACTTTTCGGGGAAATGTGCGCGGAAC<br>CCCTATTTGTTTATTTTTCTAAATACATTCAAATATGTATCCGCTCATGAATTAATTC<br>TTAGAAAAACTCATCGAGCATCAAATGAAACTGCAATTTATTCATATCAGGATTAT<br>CAATACCATATTTTTGAAAAAGCCGTTTCTGTAATGAAGGAGAAAACTCACCGAG<br>GCAGTTCCATAGGATGGCAAGATCCTGGTATCGGTCTGCGATTCCGACTCGTC<br>CAACATCAATACAACCTATTAATTTCCCCTCGTCAAAAATAAGGTTATCAAGTGAG<br>AAATCACCATGAGTGACGACTGAATCCGGTGAGAATGGCAAAAGTTTATGCATT<br>TCTTTCCAGACTTGTTCAACAGGCCAGCCATTACGCTCGTCATCAAAATCACTC<br>GCATCAACCAAACCGTTATTCATTCGTGATTGCGCCTGAGCGAGACGAAATACG<br>CGATCGCTGTTAAAAGGACAATTACAAACAGGAATCGAATGCAACCGGCGCAG<br>GAACACTGCCAGCGCATCAACAATATTTTCACCTGAATCAGGATATTCTTCTAAT<br>ACCTGGAATGCTGTTTTCCCGGGGATCGCAGTGGTGAGTAACCATGCATCATC<br>AGGAGTACGGATAAAATGCTTGATGGTCGGAAGAGGCATAAATTCCGTCAGCCA<br>GTTTAGTCTGACCATCTCATCTGTAACATCATTGGCAACGCTACCTTTGCCATGT<br>TTCAGAAACAACTCTGGCGCATCGGGCTTCCCATACAATCGATAGATTGTCGCA<br>CCTGATTGCCCGACATTATCGCGAGCCCATTTATACCCATATAAATCAGCATCCA<br>TGTTGGAATTTAATCGCGGCCTAGAGCAAGACGTTTCCCGTTGAATATGGCTCA<br>TAACACCCCTTGTATTACTGTTTATGTAAGCAGACAGTTTTATTGTTCATGACCAA<br>AATCCCTTAACGTGAGTTTTCGTTCCACTGAGCGTCAGACCCCGTAGAAAAGAT<br>CAAAGGATCTTCTTGAGATCCTTTTTTTCTGCGCGTAATCTGCTGCTTGCAAACA<br>AAAAAACCACCGCTACCAGCGGTGGTTTGTTTGCCGGATCAAGAGCTACCAAC<br>TCTTTTTCCGAAGGTAACTGGCTTCAGCAGAGCGCAGATACCAAATACTGTCCT<br>TCTAGTGTAGCCGTAGTTAGGCCACCACTTCAAGAACTCTGTAGCACCGCCTAC<br>ATACCTCGCTCTGCTAATCCTGTTACCAGTGGCTGCTGCCAGTGGCGATAAGTC<br>GTGTCTTACCGGGTTGGACTCAAGACGATAGTTACCGGATAAGGCGCAGCGGT<br>CGGGCTGAACGGGGGGTTCGTGCACACAGCCCAGCTTGGAGCGAACGACCT<br>ACACCGAACTGAGATACCTACAGCGTGAGCTATGAGAAAGCGCCACGCTTCCC<br>GAAGGGAGAAAGGCGGACAGGTATCCGGTAAGCGGCAGGGTCGGAACAGGA<br>GAGCGCACGAGGGAGCTTCCAGGGGGAAACGCCTGGTATCTTTATAGTCCTGT<br>CGGGTTTCGCCACCTCTGACTTGAGCGTCGATTTTTGTGATGCTCGTCAGGGG<br>GGCGGAGCCTATGGAAAAACGCCAGCAACGCGGCCTTTTTACGGTTCCTGGC<br>CTTTTGCTGGCCTTTTGCTCACATGTTCTTTCCTGCGTTATCCCCTGATTCTGTG<br>GATAACCGTATTACCGCCTTTGAGTGAGCTGATACCGCTCGCCGCAGCCGAAC<br>GACCGAGCGCAGCGAGTCAGTGAGCGAGGAAGCGGAAGAGCGCCTGATGCG<br>GTATTTTCTCCTTACGCATCTGTGCGGTATTTCACACCGCATATATGGTGCACTC<br>TCAGTACAATCTGCTCTGATGCCGCATAGTTAAGCCAGTATACACTCCGCTATCG<br>CTACGTGACTGGGTCATGGCTGCGCCCCGACACCCGCCAACACCCGCTGACG<br>CGCCCTGACGGGCTTGTCTGCTCCCGGCATCCGCTTACAGACAAGCTGTGAC<br>CGTCTCCGGGAGCTGCATGTGTCAGAGGTTTTCACCGTCATCACCGAAACGCG<br>CGAGGCAGCTGCGGTAAAGCTCATCAGCGTGGTCGTGAAGCGATTCACAGAT<br>GTCTGCCTGTTCATCCGCGTCCAGCTCGTTGAGTTTCTCCAGAAGCGTTAATGT<br>CTGGCTTCTGATAAAGCGGGCCATGTTAAGGGCGGTTTTTTCCTGTTTGGTCAC<br>TGATGCCTCCGTGTAAGGGGGATTTCTGTTCATGGGGGTAATGATACCGATGAA<br>ACGAGAGAGGATGCTCACGATACGGGTTACTGATGATGAACATGCCCGGTTACT<br>GGAACGTTGTGAGGGTAAACAACTGGCGGTATGGATGCGGCGGGACCAGAGA<br>AAAATCACTCAGGGTCAATGCCAGCGCTTCGTTAATACAGATGTAGGTGTTCCA<br>CAGGGTAGCCAGCAGCATCCTGCGATGCAGATCCGGAACATAATGGTGCAGGG<br>CGCTGACTTCCGCGTTTCCAGACTTTACGAAACACGGAAACCGAAGACCATTC

	ATGTTGTTGCTCAGGTCGCAGACGTTTTGCAGCAGCAGTCGCTTCACGTTCGC<br>TCGCGTATCGGTGATTCATTCTGCTAACCAGTAAGGCAACCCCGCCAGCCTAG<br>CCGGGTCCTCAACGACAGGAGCACGATCATGCGCACCCGTGGGGCCGCCATG<br>CCGGCGATAATGGCCTGCTTCTCGCCGAAACGTTTGGTGGCGGGACCAGTGA<br>CGAAGGCTTGAGCGAGGGCGTGCAAGATTCCGAATACCGCAAGCGACAGGCC<br>GATCATCGTCGCGCTCCAGCGAAAGCGGTCCTCGCCGAAAATGACCCAGAGC<br>GCTGCCGGCACCTGTCCTACGAGTTGCATGATAAAGAAGACAGTCATAAGTGC<br>GGCGACGATAGTCATGCCCCGCGCCCACCGGAAGGAGCTGACTGGGTTGAAG<br>GCTCTCAAGGGCATCGGTCGAGATCCCGGTGCCTAATGAGTGAGCTAACTTAC<br>ATTAATTGCGTTGCGCTCACTGCCCGCTTTCCAGTCGGGAAACCTGTCGTGCC<br>AGCTGCATTAATGAATCGGCCAACGCGCGGGGAGAGGCGGTTTGCGTATTGG<br>GCGCCAGGGTGGTTTTTCTTTTCACCAGTGAGACGGGCAACAGCTGATTGCCC<br>TTCACCGCCTGGCCCTGAGAGAGTTGCAGCAAGCGGTCCACGCTGGTTTGCC<br>CCAGCAGGCGAAAATCCTGTTTGATGGTGGTTAACGGCGGGATATAACATGAG<br>CTGTCTTCGGTATCGTCGTATCCCACTACCGAGATATCCGCACCAACGCGCAGC<br>CCGGACTCGGTAATGGCGCGCATTGCGCCCAGCGCCATCTGATCGTTGGCAA<br>CCAGCATCGCAGTGGGAACGATGCCCTCATTCAGCATTTGCATGGTTTGTTGAA<br>AACCGGACATGGCACTCCAGTCGCCTTCCCGTTCCGCTATCGGCTGAATTTGA<br>TTGCGAGTGAGATATTTATGCCAGCCAGCCAGACGCAGACGCGCCGAGACAGA<br>ACTTAATGGGCCCGCTAACAGCGCGATTTGCTGGTGACCCAATGCGACCAGAT<br>GCTCCACGCCCAGTCGCGTACCGTCTTCATGGGAGAAAATAATACTGTTGATGG<br>GTGTCTGGTCAGAGACATCAAGAAATAACGCCGGAACATTAGTGCAGGCAGCT<br>TCCACAGCAATGGCATCCTGGTCATCCAGCGGATAGTTAATGATCAGCCCACTG<br>ACGCGTTGCGCGAGAAGATTGTGCACCGCCGCTTTACAGGCTTCGACGCCGC<br>TTCGTTCTACCATCGACACCACCACGCTGGCACCCAGTTGATCGGCGCGAGAT<br>TTAATCGCCGCGACAATTTGCGACGGCGCGTGCAGGGCCAGACTGGAGGTGG<br>CAACGCCAATCAGCAACGACTGTTTGCCCGCCAGTTGTTGTGCCACGCGGTTG<br>GGAATGTAATTCAGCTCCGCCATCGCCGCTTCCACTTTTTCCCGCGTTTTCGCA<br>GAAACGTGGCTGGCCTGGTTCACCACGCGGGAAACGGTCTGATAAGAGACAC<br>CGGCATACTCTGCGACATCGTATAACGTTACTGGTTTCACATTCACCACCCTGA<br>ATTGACTCTCTTCCGGGCGCTATCATGCCATACCGCGAAAGGTTTTGCGCCATT<br>CGATGGTGTCCGGGATCTCGACGCTCTCCCTTATGCGACTCCTGCATTAGGAA<br>GCAGCCCAGTAGTAGGTTGAGGCCGTTGAGCACCGCCGCCGCAAGGAATGGT<br>GCATGCAAGGAGATGGCGCCCAACAGTCCCCCGGCCACGGGGCCTGCCACC<br>ATACCCACGCCGAAACAAGCGCTCATGAGCCCGAAGTGGCGAGCCCGATCTT<br>CCCCATCGGTGATGTCGGCGATATAGGCGCCAGCAACCGCACCTGTGGCGCC<br>GGTGATGCCGGCCACGATGCGTCCGGCGTAGAGGATCGAGATCTCGATCCCG<br>CGAAATTAATACGACTCACTATAGGGGAATTGTGAGCGGATAACAATTCCCCTCT<br>AGAAATAATTTTGTTTAACTTTAAGAAGGAGATATACCATGGGCAGCAGCCATCA<br>TCATCATCATCACAGCAGCGGCCTGGTGCCGCGCGGCAGCCATATGGCTAGCA<br>TGACTGGTGGACAGCAAATGGGTCGCGGATCCATGCGCGCACACTTAGACCG<br>CTTTCCAGATGCGGGCGTTGAAGGAGTCGGTGCCGCTTTAGCCCACGATGAAC<br>AAGCCGACGCAGGCCCCCACCGTGCGGTTGTAGTTGCGTCTAGCACATCGGA<br>ATTATTGGACGGATTAGCTGCGGTAGCTGACGGTCGTCCACACGCGAGTGTAG<br>TGCGTGGAGTGGCTCGTCCTTCTGCGCCTGTTGTTTTCGTGTTCCCAGGGCAG<br>GGAGCCCAGTGGGCTGGAATGGCTGGGGAGTTATTGGGGGAATCACGTGTGT<br>TCGCAGCGGCAATGGATGCTTGCGCCCGTGCCTTTGAGCCGGTAACTGATTGG<br>ACGTTGGCTCAGGTGCTTGATAGTCCCGAACAGAGTCGTCGTGTCGAAGTGGT<br>TCAACCCGCACTGTTTGCAGTTCAAACAAGTCTGGCCGCCCTGTGGCGCTCGT<br>TTGGCGTAACTCCTGATGCCGTTGTTGGGCACTCTATTGGAGAGCTTGCAGCA<br>GCACACGTGTGCGGTGCAGCGGGGGCCGCAGACGCGGCGCGCGCTGCTGC<br>CCTGTGGAGTCGCGAAATGATCCCCCTTGTCGGCAACGGCGATATGGCTGCCG<br>TAGCACTGTCCGCTGATGAAATCGAACCCCGTATTGCACGTTGGGACGATGAC<br>GTTGTCCTGGCGGGTGTTAACGGTCCGCGCTCAGTTCTGCTGACCGGATCAC<br>CGGAGCCAGTGGCCCGCCGTGTTCAGGAGTTGAGCGCTGAGGGGGTGCGTG<br>CTCAAGTTATCAACGTCAGTATGGCTGCGCATTCTGCACAAGTGGACGATATCG

	CTGAGGGTATGCGTTCGGCACTTGCATGGTTTGCCCCGGGTGGCTCAGAAGTT<br>CCTTTCTACGCGTCCCTTACGGGGGGAGCAGTAGATACGCGCGAACTGGTGG<br>CCGATTACTGGCGCCGCAGTTTTCGTCTTCCTGTGCGTTTCGATGAGGCAATC<br>CGTTCCGCTTTGGAGGTCGGGCCAGGGACATTCGTTGAAGCGAGCCCTCACC<br>CGGTTTTAGCCGCCGCCCTTCAGCAGACACTTGATGCGGAGGGTTCTAGTGCA<br>GCCGTTGTACCCACCCTACAGCGCGGACAAGGTGGCATGCGCCGCTTCCTGT<br>TAGCCGCGGCCCAAGCCTTTACTGGAGGGGTCGCAGTTGATTGGACAGCCGC<br>ATACGACGATGTGGGCGCTGAGCCCGGTTCCTTGCCAGAGTTCGCCCCAGCT<br>GAAGAAGAAGACGAACCAGCCGAATCAGGGGTCGATTGGAATGCACCCCCTC<br>ATGTATTGCGCGAACGCTTATTGGCCGTTGTAAACGGGGAGACCGCTGCCCTG<br>GCCGGTCGTGAGGCCGATGCAGAGGCTACGTTTCGCGAACTTGGATTGGACA<br>GTGTATTGGCCGCGCAACTGCGCGCGAAAGTGTCAGCGGCCATCGGGCGTGA<br>AGTCAACATTGCATTGTTGTATGATCATCCTACGCCTCGCGCATTAGCGGAGGC<br>TCTTGCTGCGGGTACCGAGGTTGCTCAGCGCGAGACACGTGCGCGTACAAAT<br>GAAGCCGCGCCGGGGGAACCGGTTGCTGTCGTCGCTATGGCGTGCCGTTTGC<br>CCGGAGGGGTCTCAACTCCGGAGGAGTTCTGGGAATTACTGTCTGAAGGACG<br>TGACGCAGTAGCTGGACTGCCTACCGATCGTGGTTGGGATTTGGATTCATTATT<br>CCACCCGGACCCCACACGCTCCGGTACTGCTCATCAACGTGGCGGGGGTTTC<br>CTGACTGAGGCGACTGCCTTTGACCCAGCCTTTTTTGGGATGTCCCCCCGTGA<br>AGCCCTTGCAGTCGATCCGCAGCAACGCTTGATGTTGGAACTTAGCTGGGAAG<br>TCTTAGAGCGCGCTGGGATTCCTCCAACTTCTCTTCAAGCATCTCCGACTGGA<br>GTTTTCGTGGGCCTTATCCCGCAAGAGTATGGGCCGCGCTTAGCTGAAGGTGG<br>AGAAGGTGTTGAAGGTTACCTTATGACTGGGACAACCACAAGTGTAGCCAGTG<br>GCCGTATCGCCTATACACTGGGTCTGGAAGGACCTGCCATCAGTGTGGATACA<br>GCGTGCTCAAGTAGTCTGGTTGCGGTTCATTTAGCCTGCCAGTCTTTGCGTCG<br>CGGAGAATCCTCATTAGCAATGGCGGGTGGCGTGACAGTTATGCCAACTCCAG<br>GTATGTTGGTTGACTTCTCGCGTATGAACTCCCTGGCCCCAGACGGCCGCTGT<br>AAGGCGTTCTCTGCTGGTGCCAATGGTTTCGGTATGGCCGAGGGTGCAGGTAT<br>GTTATTGCTGGAACGTTTGTCGGACGCCCGTCGTAATGGTCACCCCGTTCTTG<br>CAGTATTGCGTGGCACCGCTGTCAACTCGGATGGAGCCTCTAATGGCCTGTCT<br>GCACCAAATGGTCGCGCACAGGTGCGCGTAATCCAGCAAGCTCTGGCAGAGT<br>CCGGACTTGGCCCAGCGGACATTGATGCTGTGGAGGCGCATGGTACGGGCAC<br>GCGTTTAGGTGACCCGATCGAAGCACGTGCTCTGTTCGAGGCATACGGGCGT<br>GATCGCGAGCAACCTCTTCATTTGGGATCTGTGAAATCCAACCTGGGTCACAC<br>GCAGGCCGCGGCAGGTGTAGCTGGTGTCATTAAGATGGTCTTAGCGATGCGC<br>GCGGGTACGTTACCTCGCACATTACACGCGTCAGAGCGTTCCAAAGAAATTGAT<br>TGGTCTTCTGGGGCTATTAGCCTGCTTGATGAACCTGAGCCGTGGCCGGCGG<br>GCGCGCGCCCCCGCCGCGCGGGGGTAAGTTCATTTGGTATTTCGGGAACCAA<br>TGCCCATGCGATCATTGAAGAAGCCCCACAAGTTGTTGAAGGCGAACGTGTGG<br>AGGCGGGTGATGTTGTCGCGCCGTGGGTACTGTCAGCCTCGAGTGCTGAAGG<br>ACTTCGTGCACAAGCTGCACGTTTGGCCGCACACCTGCGCGAGCATCCGGGT<br>CAGGACCCACGCGACATTGCATACTCACTTGCGACAGGACGCGCAGCGTTGC<br>CACATCGCGCTGCCTTCGCCCCAGTTGATGAATCAGCAGCACTTCGTGTCCTT<br>GATGGGTTAGCGACTGGGAACGCCGACGGAGCTGCGGTGGGCACCAGTCGT<br>GCCCAACAGCGTGCTGTGTTTGTTTTCCCTGGTCAAGGTTGGCAGTGGGCAG<br>GTATGGCGGTTGATTTGCTTGATACATCTCCCGTCTTTGCGGCTGCTTTGCGTG<br>AATGCGCGGATGCTCTGGAGCCCCACCTTGATTTTGAGGTAATCCCATTCCTTC<br>GTGCCGAAGCGGCACGTCGCGAACAGGACGCCGCGCTTAGCACTGAGCGTG<br>TAGATGTGGTTCAACCCGTTATGTTCGCTGTTATGGTGAGCTTGGCATCAATGT<br>GGCGTGCGCACGGTGTGGAGCCAGCGGCAGTTATCGGTCACAGCCAGGGCG<br>AAATCGCCGCAGCATGTGTGGCTGGGGCACTGTCATTGGACGACGCAGCTCG<br>CGTGGTCGCTCTGCGTTCCCGCGTAATCGCAACAATGCCAGGCAACAAGGGTA<br>TGGCTAGTATCGCTGCGCCCGCCGGAGAAGTTCGCGCACGTATTGGAGATCGT<br>GTTGAAATTGCAGCAGTTAATGGACCTCGTTCAGTAGTAGTTGCTGGTGACAGT<br>GATGAGCTGGACCGTTTGGTTGCCTCCTGTACCACCGAGTGTATTCGCGCAAA<br>ACGCTTAGCCGTGGACTATGCTTCCCATTCTAGTCACGTTGAAACAATCCGCGA

	TGCTTTGCACGCCGAACTGGGAGAAGACTTTCATCCTTTACCAGGATTTGTGCC<br>TTTTTTCTCCACAGTAACCGGACGTTGGACCCAGCCCGATGAGTTGGACGCCG<br>GCTATTGGTATCGCAATCTTCGTCGTACTGTCCGCTTCGCGGATGCGGTCCGTG<br>CCTTAGCGGAGCAGGGGTATCGCACGTTCTTGGAAGTTTCCGCACATCCAATT<br>CTGACCGCTGCCATTGAGGAAATTGGGGATGGGAGCGGTGCTGATTTAAGCGC<br>CATTCATTCTCTGCGCCGTGGCGATGGAAGTTTGGCAGACTTCGGTGAGGCCT<br>TAAGTCGCGCCTTTGCAGCTGGGGTCGCAGTTGATTGGGAGAGCGTACATTTA<br>GGTACGGGCGCTCGCCGCGTACCCCTTCCTACCTACCCGTTCCAGCGTGAAC<br>GTGTATGGCTTGAGCCCAAACCCGTGGCTCGTCGTTCCACAGAGGTTGATGAG<br>GTTAGTGCATTACGCTATCGCATCGAATGGCGCCCTACTGGGGCTGGAGAACC<br>CGCGCGCCTTGATGGCACCTGGCTGGTGGCCAAGTATGCCGGCACAGCTGAT<br>GAAACGTCAACAGCCGCACGTGAAGCCTTGGAGAGCGCTGGAGCCCGTGTGC<br>GCGAACTGGTAGTCGATGCCCGTTGCGGCCGTGATGAACTTGCAGAGCGTTTA<br>CGCTCCGTAGGAGAGGTAGCGGGAGTTCTGAGCTTATTGGCAGTAGATGAGGC<br>TGAGCCGGAGGAAGCTCCCCTGGCCCTGGCCAGCTTGGCGGATACATTATCGT<br>TGGTGCAAGCGATGGTATCCGCGGAACTTGGGTGTCCGCTGTGGACCGTAAC<br>CGAGAGTGCGGTGGCTACTGGTCCATTCGAGCGTGTGCGTAACGCGGCCCAC<br>GGCGCGTTGTGGGGTGTAGGCCGTGTGATTGCACTGGAAAATCCAGCGGTGT<br>GGGGAGGCTTGGTGGACGTACCTGCCGGCTCAGTGGCTGAGTTAGCACGTCA<br>CCTTGCCGCAGTTGTCAGCGGTGGGGCCGGAGAGGATCAACTGGCGCTTCGC<br>GCCGATGGGGTATATGGGCGCCGCTGGGTCCGTGCCGCAGCGCCTGCCACAG<br>ACGACGAATGGAAGCCGACCGGTACTGTGCTTGTGACTGGTGGGACAGGCGG<br>GGTTGGCGGGCAAATTGCCCGCTGGTTAGCACGCCGTGGAGCCCCCCACTTG<br>CTGTTAGTTAGTCGCAGTGGACCAGACGCCGATGGCGCCGGAGAATTAGTTGC<br>TGAACTTGAGGCCCTTGGCGCTCGTACGACAGTAGCAGCCTGCGACGTCACA<br>GATCGTGAATCGGTTCGCGAATTGCTGGGCGGGATTGGCGATGATGTTCCTTT<br>GAGTGCTGTATTTCACGCAGCAGCGACTCTTGATGACGGGACCGTTGACACAT<br>TGACAGGAGAGCGTATCGAGCGTGCCAGCCGTGCCAAGGTTTTAGGGGCACG<br>CAATCTGCATGAACTTACTCGCGAGCTGGATCTGACGGCTTTTGTGCTGTTCTC<br>ATCTTTCGCGAGCGCGTTCGGTGCCCCGGGGTTGGGTGGGTATGCCCCCGGA<br>AATGCTTACCTTGATGGACTTGCCCAGCAACGCCGCAGCGATGGGTTGCCCGC<br>GACAGCCGTAGCTTGGGGAACTTGGGCCGGGTCTGGTATGGCGGAGGGACCT<br>GTGGCCGACCGTTTTCGAAGACACGGGGTGATTGAGATGCCCCCCGAAACAG<br>CCTGTCGTGCTTTGCAAAACGCACTGGACCGTGCAGAGGTGTGCCCTATCGTC<br>ATTGATGTCCGTTGGGATCGTTTTTTGTTAGCATACACCGCTCAGCGCCCAACG<br>CGTTTGTTCGATGAAATTGATGATGCGCGCCGCGCTGCTCCACAAGCGGCAGC<br>GGAACCACGCGTAGGCGCGCTGGCTTCCTTACCCGCTCCTGAACGTGAGAAG<br>GCGCTGTTCGAGTTGGTCCGTTCCCACGCTGCCGCAGTGCTTGGGCACGCCT<br>CCGCTGAACGTGTTCCTGCGGATCAGGCTTTTGCCGAGCTGGGTGTAGATTCT<br>CTGTCTGCGCTTGAATTACGTAACCGCTTGGGTGCTGCCACAGGTGTGCGCTT<br>GCCCACTACTACTGTCTTCGACCACCCAGACGTCCGTACCTTGGCGGCGCACT<br>TGGCGGCGGAGCTCGGTGGTGCTACTGGTGCGGAACAGGCTGCTCCGGCTA<br>CTACCGCTCCGGTTGACGAACCGATCGCTATCGTAGGCATGGCTTGCCGTCTG<br>CCGGGTGAAGTTGACTCTCCGGAACGTCTGTGGGAACTGATCACCTCTGGTC<br>GTGACTCTGCTGCAGAAGTACCGGACGACCGTGGTTGGGTTCCGGACGAACT<br>GATGGCTTCTGATGCTGCGGGTACCCGTCGTGCTCACGGTAACTTCATGGCTG<br>GTGCTGGTGACTTCGACGCTGCTTTCTTCGGTATCTCTCCGCGTGAAGCTCTT<br>GCTATGGACCCGCAGCAGCGTCAAGCACTGGAAACCACCTGGGAAGCTCTGG<br>AATCTGCTGGTATCCCGCCGGAAACCCTGCGTGGTTCTGACACCGGTGTTTTC<br>GTTGGTATGTCTCACCAGGGCTACGCAACCGGTCGTCCGCGTCCGGAAGACG<br>GTGTTGACGGTTACCTGCTGACCGGTAACACCGCTTCTGTTGCTTCTGGTCGT<br>ATCGCTTACGTCCTGGGCCTGGAAGGTCCGGCTCTGACCGTTGACACTGCATG<br>CTCTTCTTCTCTGGTTGCTCTGCACACCGCTTGCGGTTCTCTGCGTGACGGCG<br>ACTGCGGTCTGGCTGTGGCGGGTGGCGTTAGCGTTATGGCTGGTCCGGAAGT<br>TTTCACCGAATTCTCTCGCCAGGGCGCTCTGTCTCCGGACGGTCGTTGCAAAC<br>CGTTCTCTGACGAAGCTGACGGTTTCGGTCTGGGTGAAGGTTCTGCTTTCGTT

GTTCTGCAGCGTCTGTCTGACGCTCGTCGTGAAGGTCGTCGTGTTTTAGGTGT
TGTTGCAGGTTCTGCTGTTAACCAGGACGGTGCTTCTAACGGTCTGTCTGCTC
CGAGCGGTGTTGCTCAGCAGCGTGTTATCCGTCGTGCTTGGGCTCGTGCTGG
CATCACTGGTGCTGACGTTGCTGTTGTTGAAGCTCACGGTACCGGTACACGTC
TGGGTGACCCGGTTGAAGCTTCTGCTCTGCTGGCTACCTACGGTAAATCTCGT
GGTTCTTCTGGTCCGGTACTGCTGGGTTCTGTTAAATCTAACATCGGTCACGCC
CAGGCTGCTGCTGGTGTTGCTGGTGTTATCAAAGTTCTGCTGGGTCTGGAACG
CGGTGTGGTTCCGCCGATGCTGTGCCGTGGTGAACGTTCTGGTCTGATCGACT
GGTCTTCTGGTGAAATCGAACTTGCTGACGGTGTACGTGAATGGTCTCCGGCT
GCAGATGGTGTTCGTCGTGCTGGCGTGTCTGCTTTCGGTGTTTCTGGTACCAA
CGCTCACGTTATCATCGCTGAACCGCCGGAACCGGAGCCGGTACCGCAGCCG
CGTCGTATGCTGCCGGCTACCGGTGTTGTACCGGTTGTTCTGTCTGCTCGTAC
CGGTGCGGCCCTGCGCGCTCAAGCAGGTCGTCTGGCTGACCACCTGGCTGC
TCACCCGGGTATCGCTCCGGCTGACGTTTCTTGGACCATGGCTCGTGCTCGTC
AGCACTTCGAAGAACGTGCCGCTGTTCTTGCGGCTGACACCGCTGAAGCTGTT
CACCGTCTGCGTGCTGTAGCTGACGGTGCAGTAGTTCCGGGTGTTGTTACCGG
TTCTGCTTCTGACGGTGGTTCTGTTTTCGTTTTCCCGGGTCAGGGTGCTCAGT
GGGAAGGTATGGCTCGTGAACTGCTGCCGGTTCCGGTTTTCGCAGAATCCATC
GCTGAATGCGACGCTGTTCTGTCTGAAGTTGCTGGTTTTTCCGTTTCTGAAGTT
CTGGAACCGCGTCCGGACGCTCCGTCTCTGGAGCGTGTTGACGTTGTTCAGC
CGGTCCTGTTTGCTGTTATGGTTTCTCTGGCTCGTCTGTGGCGTGCTTGCGGT
GCTGTGCCGTCTGCTGTTATCGGTCACTCTCAGGGTGAAATCGCTGCTGCTGT
TGTAGCAGGTGCTCTGTCTCTGGAAGACGGTATGCGTGTTGTTGCTCGTCGTT
CTCGTGCAGTTCGTGCTGTTGCTGGTCGTGGCTCTATGCTGTCTGTTCGCGGT
GGTCGTTCTGACGTTGAAAAACTGCTGGCTGACGACTCTTGGACCGGTCGTCT
GGAAGTTGCTGCTGTTAACGGTCCGGACGCTGTAGTTGTTGCTGGCGACGCTC
AGGCTGCTCGTGAATTCCTGGAATACTGCGAAGGTGTTGGCATCCGTGCTCGT
GCTATCCCGGTTGACTACGCTTCTCACACCGCTCACGTTGAACCGGTTCGCGA
TGAACTCGTTCAGGCACTGGCTGGTATCACCCCGCGTCGTGCTGAAGTTCCGT
TCTTCTCTACCCTGACCGGTGACTTCCTTGACGGTACCGAATTGGACGCTGGT
TACTGGTACCGTAACCTGCGTCACCCGGTTGAATTCCACTCTGCTGTTCAGGCT
CTGACCGACCAGGGTTACGCTACCTTCATCGAAGTTTCTCCGCACCCGGTTCT
GGCTTCTTCTGTTCAGGAAACCCTGGACGACGCTGAATCTGACGCGGCTGTAC
TTGGTACCCTGGAACGTGACGCTGGTGACGCTGACCGTTTCCTGACCGCTCT
GGCTGACGCTCACACTCGCGGCGTTGCTGTTGACTGGGAAGCTGTTCTGGGT
CGTGCAGGCCTGGTAGACCTGCCAGGTTACCCGTTCCAGGGTAAACGTTTCTG
GTTGCTGCCGGACCGTACCACCCCGCGTGACGAACTGGACGGTTGGTTCTAC
CGTGTTGACTGGACCGAAGTTCCGCGTTCTGAACCGGCTGCTCTGCGTGGTC
GTTGGCTGGTTGTTGTTCCGGAAGGTCACGAAGAAGACGGTTGGACCGTTGA
AGTTCGTTCTGCGCTGGCAGAAGCTGGTGCTGAACCGGAAGTTACCCGTGGT
GTTGGTGGTCTGGTTGGTGACTGCGCTGGTGTTGTTTCTCTGCTGGCTCTGGA
AGGTGACGGTGCTGTTCAGACCCTGGTTCTGGTTCGTGAGCTGGATGCTGAA
GGTATCGACGCTCCACTGTGGACCGTTACCTTCGGTGCTGTTGACGCTGGTTC
TCCGGTTGCTCGTCCGGACCAGGCTAAACTGTGGGGTCTGGGTCAGGTTGCT
TCTCTGGAACGTGGTCCGCGTTGGACCGGTCTGGTTGACCTGCCGCACATGC
CGGACCCGGAACTGCGTGGTCGTCTGACCGCTGTTCTGGCTGGTTCTGAAGA
CCAGGTTGCTGTACGTGCTGACGCTGTTCGTGCTCGTCGTCTGTCTCCGGCTC
ACGTTACCGCTACCTCTGAATACGCTGTTCCGGGTGGTACCATCCTGGTTACCG
GTGGTACCGCTGGTCTGGGTGCTGAAGTTGCTCGTTGGCTGGCTGGTCGTGG
TGCTGAACACCTGGCTCTGGTTTCTCGTCGTGGTCCGGACACCGAAGGTGTT
GGTGACCTGACCGCTGAATTGACCCGTCTGGGTGCTCGTGTTTCTGTTCACGC
TTGCGACGTTTCTTCTCGTGAACCGGTTCGTGAACTTGTACACGGTCTGATCGA
ACAGGGTGACGTTGTTCGTGGTGTTGTTCACGCTGCTGGCCTGCCGCAGCAG
GTTGCTATCAACGACATGGACGAAGCTGCTTTCGACGAAGTTGTTGCTGCTAAA
GCTGGTGGTGCTGTTCACCTGGACGAACTGTGCTCTGACGCAGAATTGTTCCT
GCTGTTCTCTTCTGGTGCTGGTGTTTGGGGTTCTGCTCGTCAGGGTGCTTACG

	CTGCTGGTAACGCTTTTCTGGACGCTTTCGCTCGTCACCGTCGTGGTCGTGGT<br>CTGCCGGCTACCTCTGTTGCTTGGGGTCTGTGGGCTGCTGGTGGTATGACCG<br>GTGACGAAGAAGCTGTTTCTTTCCTGCGTGAACGTGGTGTTCGCGCGATGCCG<br>GTTCCGCGTGCTCTGGCTGCTCTGGACCGTGTTCTGGCTTCTGGTGAAACCG<br>CTGTTGTTGTTACCGACGTTGACTGGCCGGCTTTCGCTGAATCTTACACCGCT<br>GCTCGTCCGCGTCCGCTGCTGGACCGTATCGTTACCACCGCTCCGTCTGAAC<br>GTGCTGGTGAACCGGAAACCGAATCTCTGCGTGACCGTCTGGCGGGCCTGCC<br>GCGTGCTGAACGTACCGCTGAACTGGTTCGTCTGGTTCGTACCTCTACCGCTA<br>CCGTTCTGGGTCACGACGACCCGAAAGCTGTTCGTGCTACCACCCCGTTCAAA<br>GAACTGGGTTTCGACTCTCTGGCTGCTGTTCGTCTGCGTAACCTGCTGAACGC<br>TGCTACCGGTCTGCGTCTGCCGTCTACCCTGGTTTTCGACCACCCGAACGCTT<br>CTGCTGTTGCTGGTTTCCTGGACGCTGAACTGTCTAGTGGCACCCCGGCGCG<br>GGAAGCGTCTAGTGCTCTGCGCGACGGGTATCGTCAGGCTGGCGTGTCGGGG<br>CGCGTACGCAGTTACTTGGATCTCCTGGCAGGTCTTTCCGACTTCCGCGAGCA<br>TTTCGATGGTTCTGATGGCTTTAGCCTTGACCTGGTGGATATGGCCGATGGTCC<br>AGGCGAAGTGACGGTCATCTGCTGTGCGGGGACCGCGGCCATTTCAGGCCCG<br>CACGAGTTTACTCGTCTCGCTGGCGCATTGCGCGGCATTGCTCCTGTGCGTGC<br>AGTTCCGCAACCAGGCTATGAGGAAGGCGAACCACTGCCGAGCAGCATGGCC<br>GCCGTGGCCGCGGTGCAGGCTGATGCAGTCATTCGCACCCAAGGTGACAAAC<br>CTTTCGTGGTAGCAGGCCACAGCGCCGGCGCACTCATGGCCTATGCACTCGC<br>GACCGAGCTGTTGGATCGTGGTCACCCGCCACGCGGGGTTGTCCTGATTGAT<br>GTATACCCGCCGGGCCACCAAGACGCTATGAACGCCTGGCTCGAAGAATTGAC<br>CGCCACGTTATTTGACCGTGAGACCGTACGCATGGACGACACTCGCTTGACCG<br>CGCTGGGTGCGTACGACCGCCTGACAGGTCAGTGGCGTCCGCGCGAAACGG<br>GTCTGCCGACACTTCTGGTGTCTGCGGGCGAACCTATGGGCCCATGGCCGGA<br>TGATTCGTGGAAACCGACCTGGCCGTTTGAGCATGACACAGTGGCTGTCCCAG<br>GCGACCATTTCACGATGGTTCAGGAACACGCCGATGCGATTGCTCGTCATATC<br>GACGCCTGGCTTGGAGGCGGGAATTCGAGCTCCGTCGACAAGCTTGCGGCC<br>GCACTCGAGCACCACCACCACCACCACTGAGATCCGGCTGCTAACAAAGCCC<br>GAAAGGAAGCTGAGTTGGCTGCTG

Supplementary Table S3. *E. coli* strains used in this study.

Bacterial Strain	Genotype/ Description	Reference
E. coli TOP10	F- mcrA Δ(mrr-hsdRMS-mcrBC) φ80lacZΔM15 ΔlacX74 recA1 araD139 Δ(ara-leu)7697 galU galK λ- rpsL(StrR) endA1 nupG	Invitrogen
E. coli BL21 (DE3)	F¯ ompT gal dcm lon hsdSB(rB-mB-) λ(DE3 *lacI lacUV5-T7 gene 1 ind1 sam7 nin5]	Invitrogen
E. coli K207-3	F^- *ompT hsdS* (r_B^- m_B^-) *gal dcm* (DE3) *panD*::*panDS2SA*D*prpRBCD*::T7 prom-*sfp* T7 prom-*prpE ygfG*::T7 prom-*accA1*-T7 prom-*pccB* DTolC	Murli, S.; Kennedy, J.; Dayem, L. C.; Carney, J. R.; Kealey, J. T. Metabolic Engineering of Escherichia Coli for Improved 6-Deoxyerythronolide B Production. *J Industrial Microbiol Biotechnology* 2003, *30* (8), 500–509

Supplementary Table S4. Oligonucleotides used in this study.

Entry	Name	Sequence	Function
1	AcsA_Fwd	CCCCTCAAGACCCGTTTAGAGGC	Amplification of WT AcsA during error prone PCR
2	AcsA_Rev	GCCATCATCATCATCATCACAGCAGC	Amplification of WT AcsA during error prone PCR
3	pEt28a_Fwd	GAGATCGCCAAACAGCAGGCCCTG	Amplification of pEt28a backbone
4	pET28a_Rev	CACCACGTGCTCATAGTCCC	Amplification of pEt28a backbone
5	G417X_Fwd	CTACTACCTGAGCnnkGACATCGTGGAG	Saturation Library
6	G417X_Rev	CGGCCGGCGAAGGCC	Saturation Library
7	R534X_Fwd	CATCCTGnnkAACCAGGAGATCGC	Saturation Library
8	R534X_Rev	AACCGCTGCAGTTTGCCGCTC	Saturation Library
9	D418X_Fwd	CCTGAGCGGCGACATCGTGGAGC	Saturation Library
10	D418X_Rev	TAGTAGCGGCCGGCGAAGGCCTG	Saturation Library
11	F430X_Fwd	CATCAGCnnkGTCGGCCGCAACGAC	Saturation Library
12	F430X_Rev	CTGCCGTCGTCGTTCAGCTCC	Saturation Library
13	pCDF_Fwd	GAGGGGTTTTTTGCTGAAAGGAAACCTCAGGCATTTGAGA	pCDFduet-AcsA
14	pCDF_Rev	AAAGTTAAACAAAATTATTATATCTCCTTATTAAAGTTAAACAAAATTATTTCTACAGGG	pCDFduet-AcsA
15	AcsA_Fragment_Fwd	AACTTTAATAAGGAGATATAATAATTTTGTTTAACTTTAAGAAGGAGATATACCATGG	pCDFduet-AcsA
16	AcsA_Fragment_Rev	TCTCAAATGCCTGAGGTTTCCTTTCAGCAAAAAACCCCTC	pCDFduet-AcsA

Supplementary Table S5. HPLC and LCMS analysis of AcsA double and triple mutants with alternative thiols.

		Thiol Substrate	Product			
Construct	Thiol/ Product	Peak Area	Calc. Mass $[M+H]^+$	Obs. Mass $[M+H]^+$	Δ Mass (Calc. – Obs.)	Peak Area
WT	**1/2** [a]	40,000	ND	ND	ND	801,000
	**3/4**	3,649,000	335.1	335.1	0	79,000
	**5/6**	953,000	176.1	176.2	0.1	2,900
D449E	**1/2** [a]	19,000	ND	ND	ND	805,000
	**3/4**	3,233,000	335.1	335.1	0	520,000
	**5/6**	929,000	176.1	176.2	0.1	43,000
L250M/ D449E	**1/2** [a]	29,000	ND	ND	ND	799,000
	**3/4**	3,236,000	335.1	335.1	0	728,000
	**5/6**	949,000	176.1	176.2	0.1	74,000
R302S/ D449E	**1/2** [a]	86,000	ND	ND	ND	749,000
	**3/4**	3,426,000	335.1	335.1	0	671,000
	**5/6**	960,000	176.1	176.2	0.1	104,000
F430V/ D449E	**1/2** [a]	67,000	ND	ND	ND	765,000
	**3/4**	3,286,000	335.1	335.1	0	1,165,000
	**5/6**	936,000	176.1	176.2	0.1	125,000
F430W/ D449E	**1/2** [a]	104,000	ND	ND	ND	1,361,000
	**3/4**	3,203,000	335.1	335.1	0	1,393,000
	**5/6**	1,009,000	176.1	176.2	0.1	143,000
L250M/ R302S/ D449E	**1/2** [a]	474,000	ND	ND	ND	1,545,000
	**3/4**	3,805,000	335.1	335.1	0	150,000
	**5/6**	1,068,000	176.1	176.2	0.1	15,000
L250M/ F430V/ D449E	**1/2** [a]	304,000	ND	ND	ND	1,221,000
	**3/4**	3,613,000	335.1	335.1	0	890,000
	**5/6**	889,000	176.1	176.2	0.1	100,000
L250M/ F430W/ D449E	**1/2** [a]	678,000	ND	ND	ND	1,756,000
	**3/4**	3,565,000	335.1	335.1	0	709,000
	**5/6**	931,000	176.1	176.2	0.1	66,000
R302S/ F430V/ D449E	**1/2** [a]	49,000	ND	ND	ND	1,631,000
	**3/4**	3,531,000	335.1	335.1	0	646,000
	**5/6**	923,000	176.1	176.2	0.1	86,000
R302S/ F430W/ D449E	**1/2** [a]	168,000	ND	ND	ND	1,553,000
	**3/4**	4,325,000	335.1	335.1	0	406,000
	**5/6**	1,283,000	176.1	176.2	0.1	60,000

[a] Reactions that utilized CoA as the thiol of interest were analyzed via HPLC using methods described in the Supplemental Methods section

[b] Reactions that utilized SNAC or Pantetheine as the thiol of interest were analyzed via LCMS according to the method described in the Supplemental Methods.

[c] Percent conversion was calculated by dividing the peak area of the acyl-thiol product by the combined peak areas of the acyl-thiol product and unreacted thiol.

Supplementary Table S6. HPLC and LCMS analysis of select AcsA mutants with various acids.

Mutant	Acid Substrate	Thiol Substrate		Product					
		Thiol	Peak Area	Ret. Time (min)	Calc. Mass $[M+H]^+$	Obs, Mass $[M+H]^+$	Δ Mass (Calc. – Obs.)	Peak Area	% Conversion[c]
D449E	**7**	CoA[a]	18,000	22.8	ND	ND	ND	2,600	12.3 ± 1.3
	**4**	CoA[a]	500	24.1	ND	ND	ND	21,000	97.7 ± 1.2
	**5**	CoA[a]	500	26.4	ND	ND	ND	20,000	96.7 ± 1.6
	**6**	CoA[a]	1,000	27.1	ND	ND	ND	19,000	95.6 ± 3.2
	**8**	CoA[a]	16,000	23.2	ND	ND	ND	8,000	30.4 ± 3.5
	**9**	CoA[a]	18,000	28.1	ND	ND	ND	4,000	15.8 ± 1.1
	**10**	CoA[a]	14,000	23.5	ND	ND	ND	3,000	14.5 ± 1.5
F430W / D449E	**7**	CoA[a]	17,000	22.8	ND	ND	ND	2,000	8.6 ± 1.5
	**4**	CoA[a]	1,000	24.1	ND	ND	ND	12,000	92.2 ± 0.7
	**5**	CoA[a]	1,000	26.4	ND	ND	ND	10,000	95.2 ± 3.1
	**6**	CoA[a]	1,000	27.1	ND	ND	ND	19,000	96.1 ± 1.4
	**8**	CoA[a]	13,000	23.2	ND	ND	ND	1,000	10.2 ± 2.3
	**9**	CoA[a]	12,000	28.1	ND	ND	ND	7,000	36.6 ± 2.4
	**10**	CoA[a]	ND	ND	ND	ND	ND	ND	ND
D449E	**7**	SNAC[b]	821,000	3.78	162.1	162.1	0	13,000	1.6 ± 0.1
	**4**	SNAC[b]	610,000	4.15	176.1	176.2	0.1	2,000	3.9 ± 0.7
	**5**	SNAC[b]	672,000	4.45	190.1	190.1	0	12,000	1.93 ± 0.2
	**6**	SNAC[b]	659,000	4.53	190.1	190.1	0	165,000	20.6 ± 0.6
	**8**	SNAC[b]	710,000	4.34	200.1	200.2	0.1	1,000	1.5 ± 0.3
	**9**	SNAC[b]	705,000	4.73	244.1	244.1	0	16,000	2.7 ± 0.4
	**10**	SNAC[b]	720,000	4.43	218.1	218.2	0.1	3,000	3.7 ± 0.9
F430W / D449E	**7**	SNAC[b]	650,000	3.78	162.1	162.1	0	19,000	2.9 ± 0.2
	**4**	SNAC[b]	670,000	4.15	176.1	176.2	0.1	90,000	13.2 ± 0.5
	**5**	SNAC[b]	638,000	4.45	190.1	190.1	0	20,000	2.9 ± 0.3
	**6**	SNAC[b]	643,000	4.53	190.1	190.1	0	305,000	30.5 ± 1.6
	**8**	SNAC[b]	610,000	4.34	200.1	200.2	0.1	16,000	2.5 ± 0.5
	**9**	SNAC[b]	685,000	4.73	244.1	244.1	0	58,000	7.7 ± 0.2
	**10**	SNAC[b]	ND	ND	ND	ND	ND	ND	ND

[a] Reactions that utilized CoA as the thiol of interest were analyzed via HPLC using methods described in the Supplemental Methods section

[b] Reactions that utilized SNAC as the thiol of interest were analyzed via LCMS according to the method described in the Supplemental Methods.

[c] Percent conversion was calculated by dividing the peak area of the acyl-thiol product by the combined peak areas of the acyl-thiol product and unreacted thiol.

Supplementary Table S7. LCMS analysis of *in situ* pyrone production assays.

Conditions Set[a]	Retention Time (min)	Calc. Mass $[M+H]^+$	Obs. Mass $[M+H]^+$	Δ Mass (Calc. – Obs.)	Peak Area	Calculated Pyrone Concentration (μM)
1	4.42	169.1	169.2	0.1	118,000 ± 23,000	28.2
2	4.42	169.1	169.2	0.1	190,000 ± 12,000	51.8
3	4.42	169.1	169.2	0.1	282,000 ± 8,000	82.7
4	4.42	169.1	169.2	0.1	279,000 ± 35,000	81.5
5	4.42	169.1	169.2	0.1	204,000 ± 34,000	56.7
6	4.42	169.1	169.2	0.1	252,000 ± 14,000	72.6
7	4.42	169.1	169.2	0.1	219,000 ± 19,000	61.6
8	4.42	169.1	169.2	0.1	1,674,000 ± 152,000	545.2
9	4.42	169.1	169.2	0.1	246,000 ± 36,000	70.5
10	4.42	169.1	169.2	0.1	392,000 ± 28,000	118.9
11	4.42	169.1	169.2	0.1	1,021,000 ± 68,000	328.5
12	4.42	169.1	169.2	0.1	1,242,000 ± 65,0000	401.7
15.625 μM TAL	3.65	127.1	127.1	0	1,016,000 ± 48,000	ND
31.25 μM TAL	3.65	127.1	127.1	0	1,918,000 ± 14,000	ND
61.125 μM TAL	3.65	127.1	127.1	0	3,697,000 ± 145,0000	ND

[a] See Figure 6 for description of each condition

Supplemental Figures

Supplementary Figure S1. Plasmid map of pET28a-AcsA.

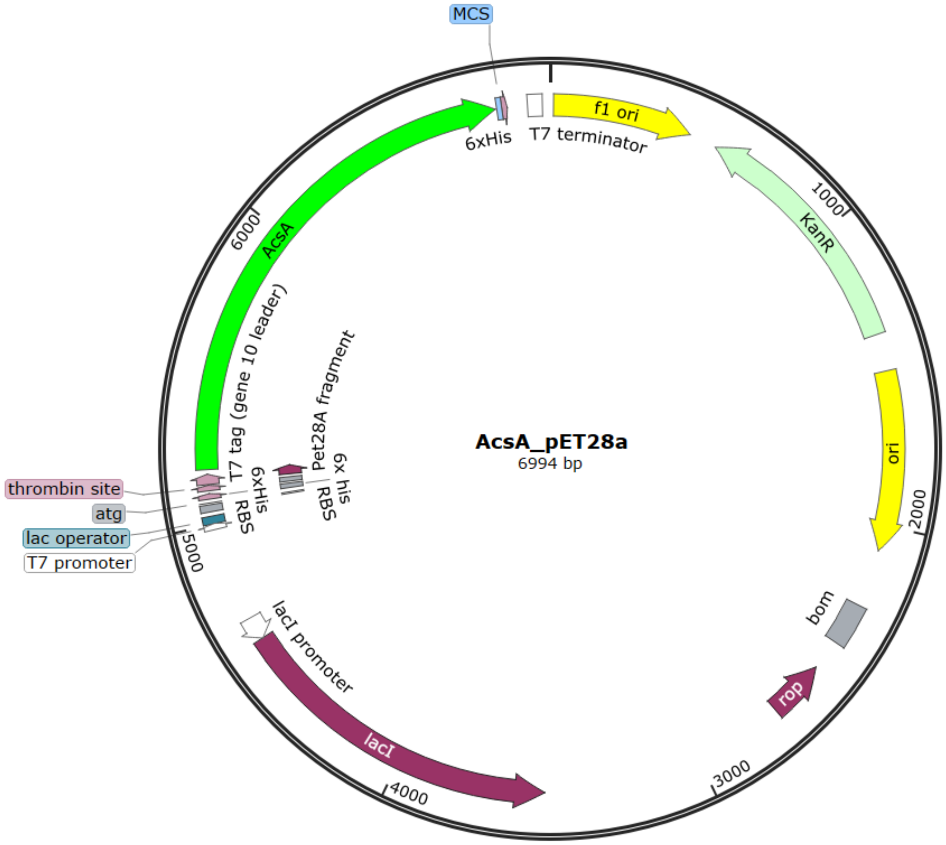

Supplementary Figure S2. SDS–PAGE analysis of purified AcsA variants. Purified AcsA proteins were analyzed by 15% SDS–PAGE to assess expression and purity. Lane 1, PageRuler™ prestained protein ladder (Thermo Fisher Scientific). Lane 2, WT AcsA. Lane 3, AcsA D449E. Lane 4, AcsA L250M/D449E. Lane 5, AcsA R302S/D449E. Lane 6, AcsA F430V/D449E. Lane 7, AcsA F430W/D449E. Lane 8, AcsA L250M/R302S/D449E. Lane 9, AcsA L250M/F430V/D449E. Lane 10, AcsA L250M/F430W/D449E. Lane 11, AcsA R302S/F430V/D449E. Lane 12, AcsA R302S/F430W/D449E. A band corresponding to the expected molecular weight of AcsA (60.2 kDa) is observed for all purified variants.

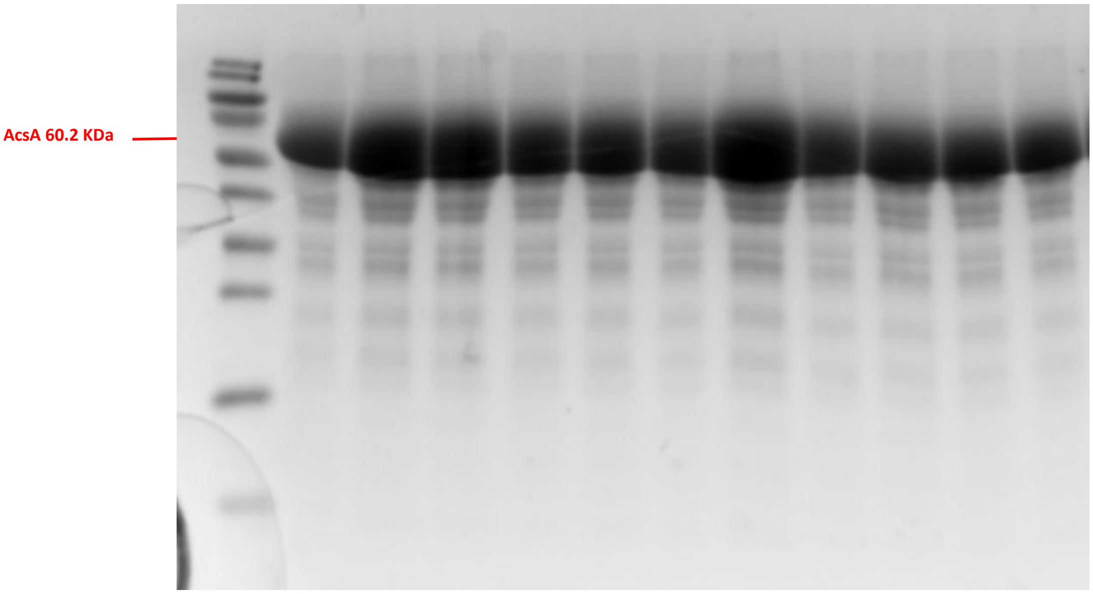

Supplementary Figure S3. Time course assays for various AcsA mutants with different thiols were used to estimate the apparent initial reaction rates

Supplementary Figure S3-1. CoA Time Course Assay with Various AcsA Mutants.

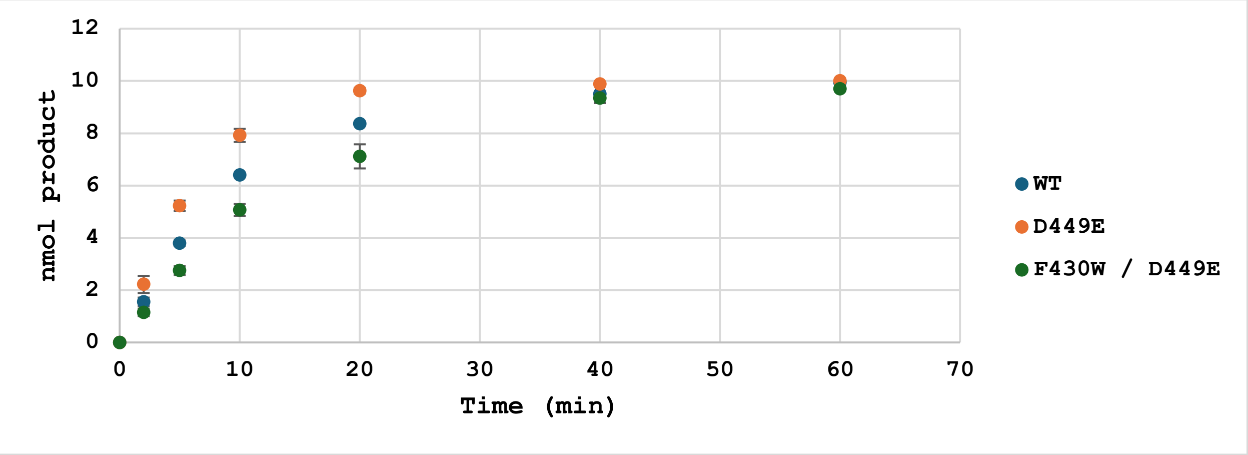

Supplementary Figure S3-2. (R)-Pantetheine Time Course Assay with Various AcsA Mutants.

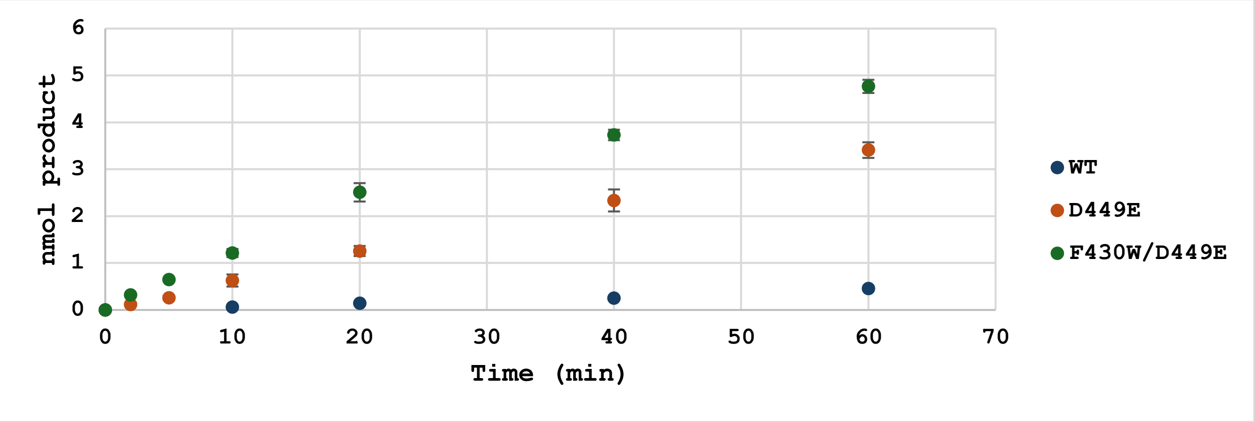

Supplementary Figure S3-3. HSNAC Time Course Assay with Various AcsA Mutants.

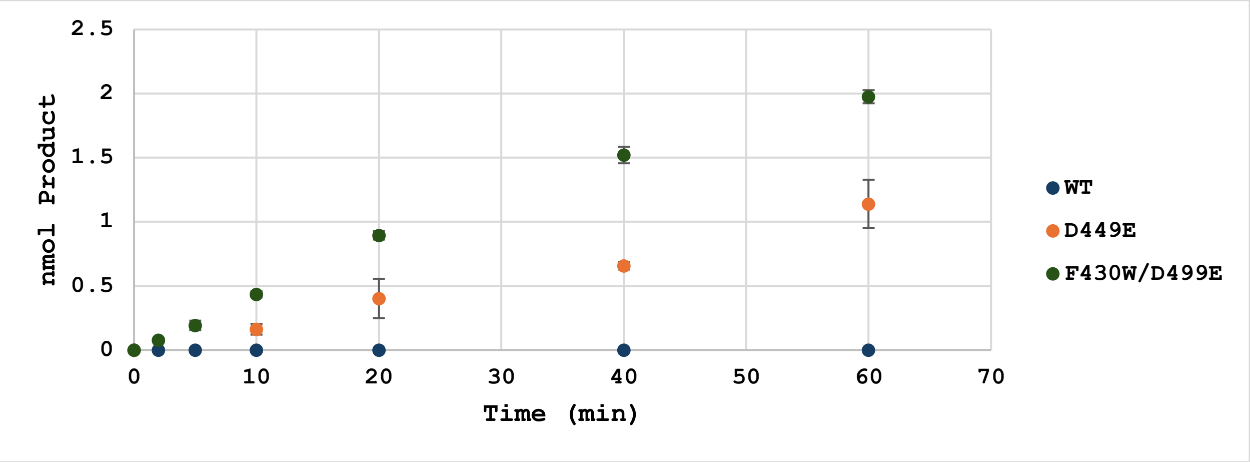

Supplementary Figure S4. Representative LC–MS extracted ion chromatograms (XICs) from AcsA carboxylic acid panel assays. Representative XIC traces corresponding to the expected *m/z* values of acyl-SNAC products formed during LC–MS analysis are shown. A representative SNAC peak is included to indicate the characteristic retention time of the free thiol under the analytical conditions used.

(A) SNAC.

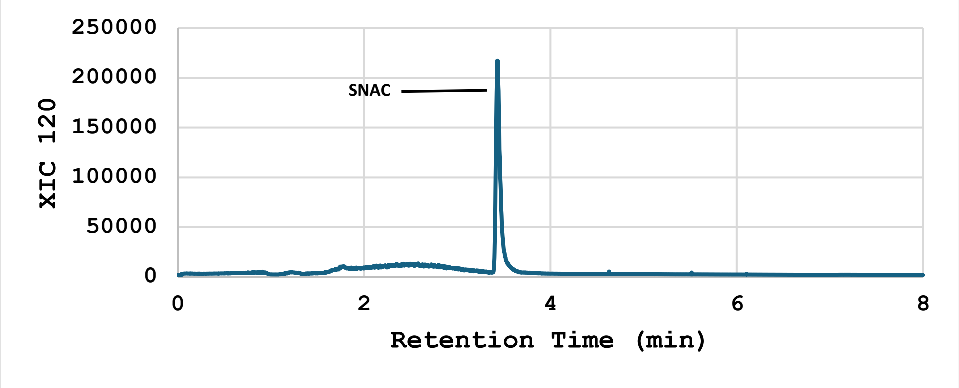

(B) Acetyl-SNAC.

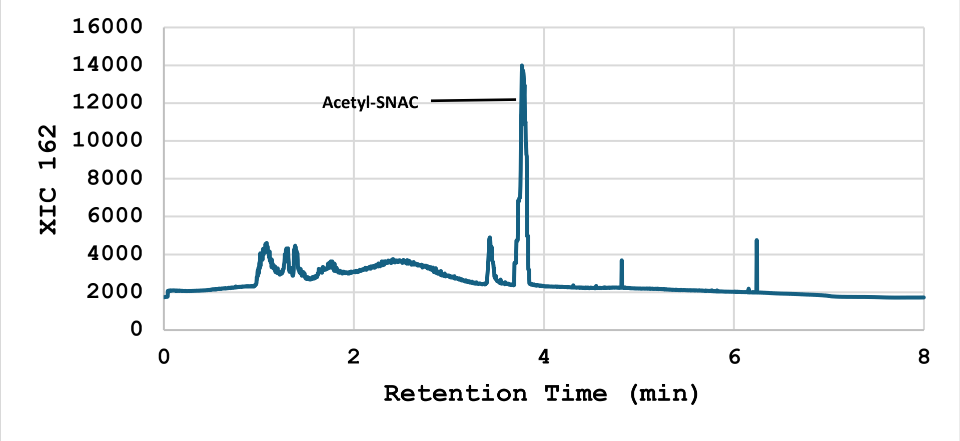

(C) Propionyl-SNAC.

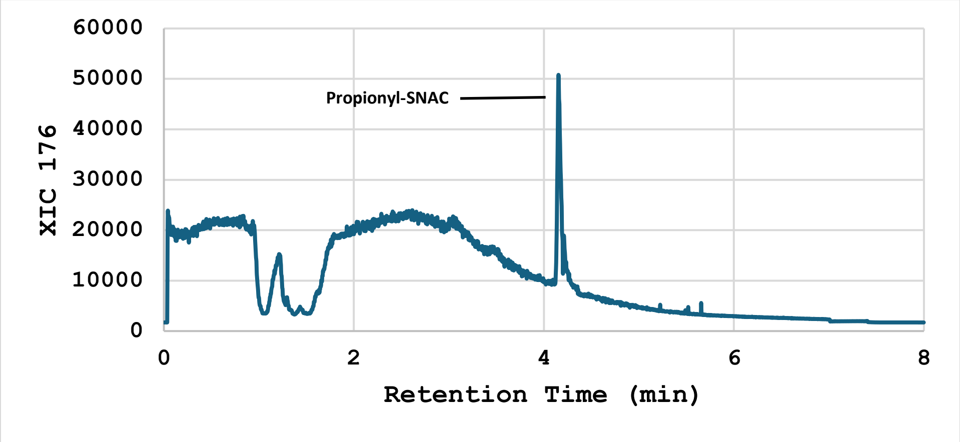

(D) Butyryl-SNAC.

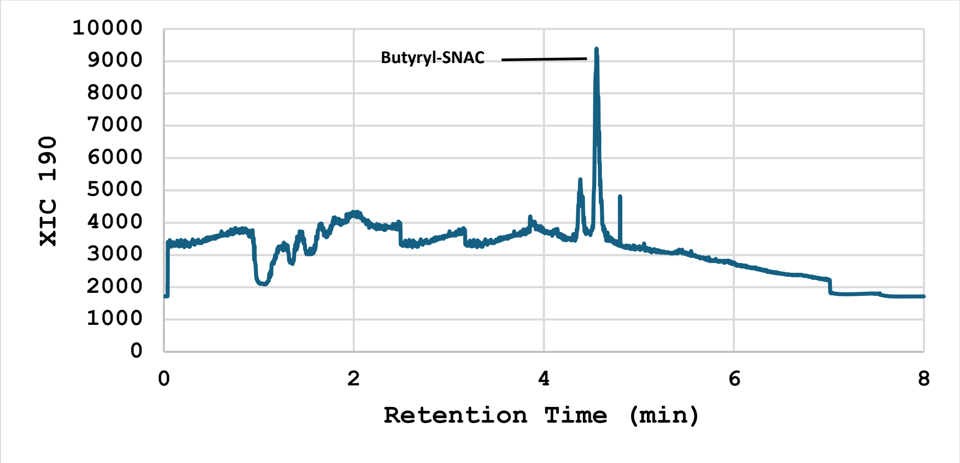

(E) *iso*-Butyryl-SNAC.

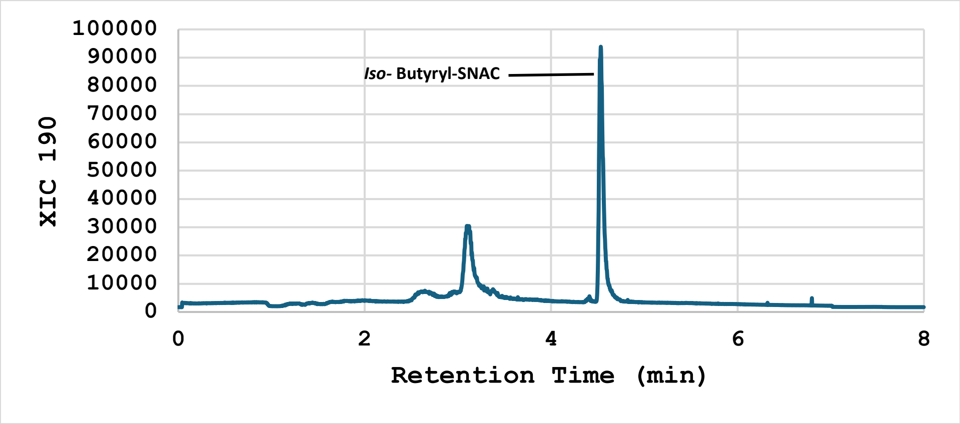

(F) 4-Pentanoyl-SNAC.

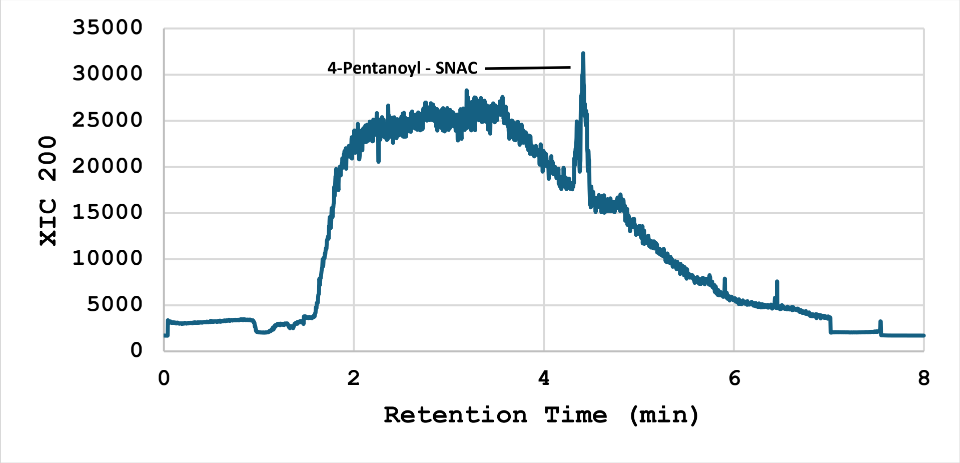

(G) 4,4,4-Trifluorobutyryl-SNAC.

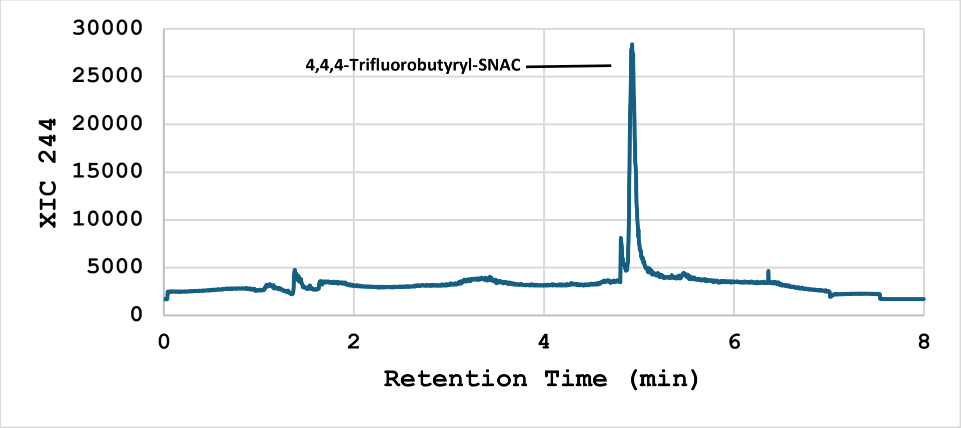

Supplementary Figure S5. Plasmid maps of the two-plasmid system used for *in situ* pyrone production.

Supplementary Figure S5-1. Plasmid Map of pCDFDuet-AcsA

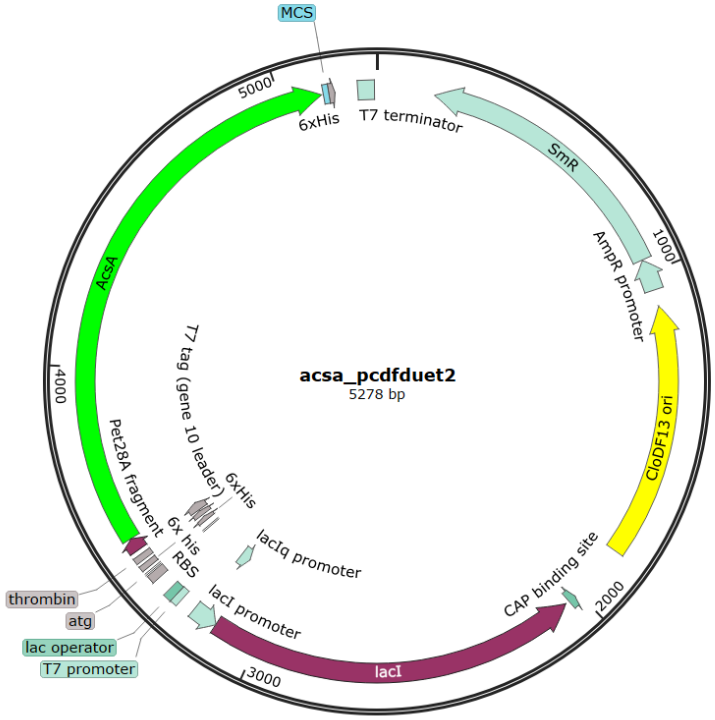

Supplementary Figure S5-2. Plasmid Map of pCDFDuet-AcsA

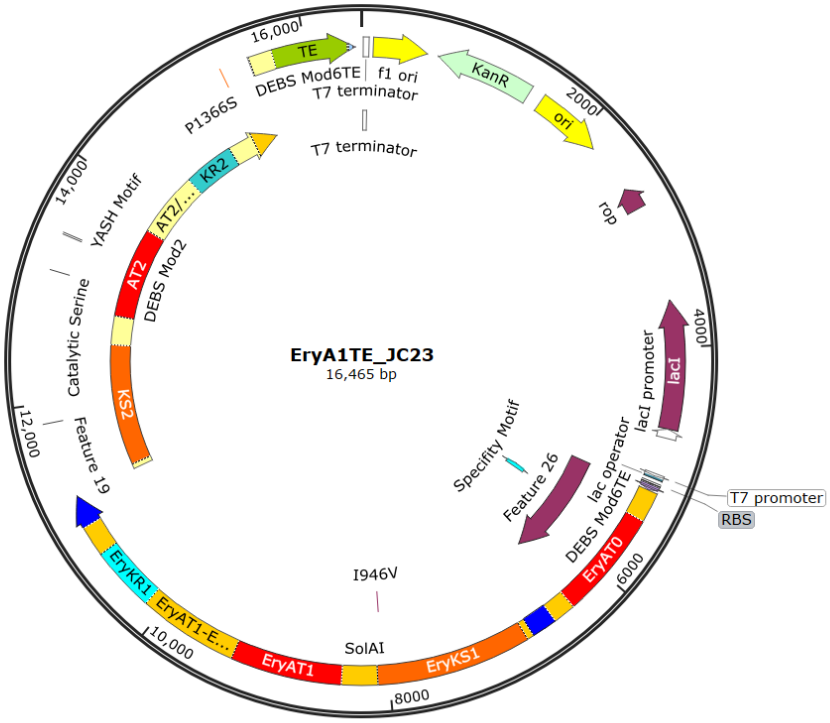

Supplementary Figure S6. SDS page gels enzymes used for *in situ* pyrone production.

Supplementary Figure S6-1. SDS–PAGE analysis of AcsA expression in clarified cell lysates. Clarified lysates from *E. coli* cultures expressing the indicated constructs were analyzed by 15% SDS–PAGE to confirm protein expression. Lane 1, PageRuler™ prestained protein ladder (Thermo Fisher Scientific). Lane 2, double empty vector negative control (empty pCDFDuet and empty pET28a). Lane 3, lysate from cells harboring $AcsA_{DEFW}$-pCDFDuet and empty pET28a. Lane 4, lysate from cells harboring empty pCDFDuet and EryA1TE-pET28a. Lane 5, lysate from cells harboring WT AcsA-pCDFDuet and EryA1TE-pET28a. Lane 6, lysate from cells harboring $AcsA_{DEFW}$-pCDFDuet and EryA1TE-pET28a. A band corresponding to the expected molecular weight of AcsA (60.2 kDa) is observed in lanes 3, 5, and 6 and is absent in the negative control lanes (lanes 2 and 4).

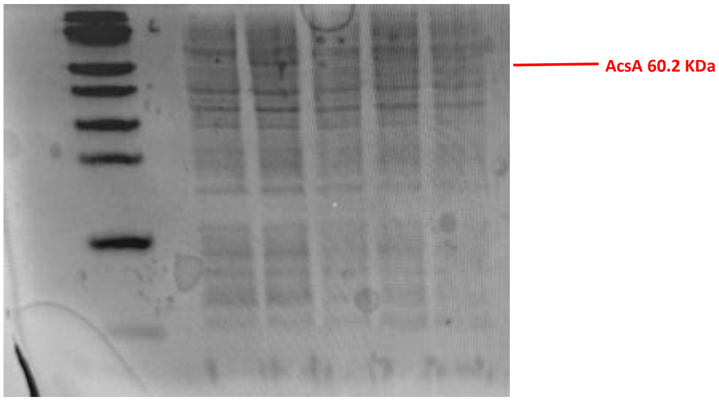

Supplementary Figure S6-2. SDS–PAGE analysis of EryA1TE expression in clarified cell lysates. Clarified lysates from *E. coli* cultures expressing the indicated constructs were analyzed by 15% SDS–PAGE to confirm protein expression. Lane 1, HiMark™ prestained high–molecular weight protein ladder (Thermo Fisher Scientific). Lane 2, double empty vector negative control (empty pCDFDuet and empty pET28a). Lane 3, lysate from cells harboring $AcsA_{DEFW}$-pCDFDuet and empty pET28a. Lane 4, lysate from cells harboring empty pCDFDuet and EryA1TE-pET28a. Lane 5, lysate from cells harboring WT AcsA-pCDFDuet and EryA1TE-pET28a. Lane 6, lysate from cells harboring $AcsA_{DEFW}$-pCDFDuet and EryA1TE-pET28a. A band corresponding to the expected molecular weight of EryA1TE (392 kDa) is observed in lanes 4–6 and is absent in lanes 2 and 3.

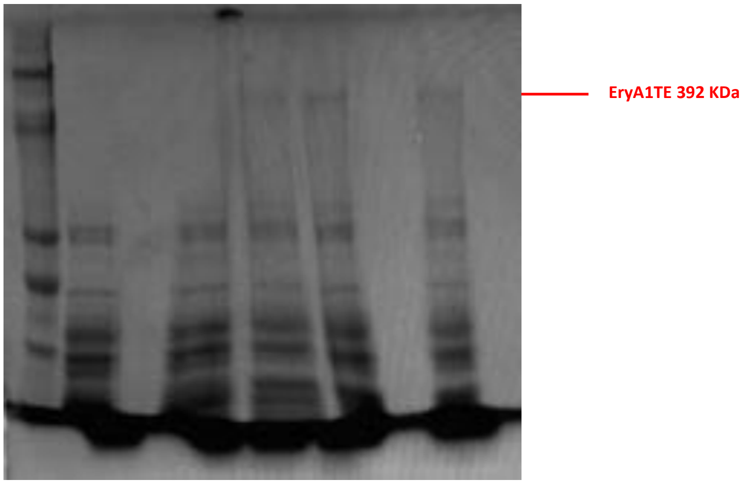

Supplementary Figure S7. Representative LCMS chromatogram demonstrating *in situ* pyrone formation with calibration curve for internal standard TAL. Left: XICs for the calibration curve samples of TAL at various concentrations as well as a representative trace from the 4 mM SNAC reaction subset. Right: Plotted calibration curve of TAL at various concentrations demonstrates a linear relationship (R^2 of 0.999) around the concentrations of TAL used to determine pyrone concentration.

(A) XIC 312.5 µM TAL

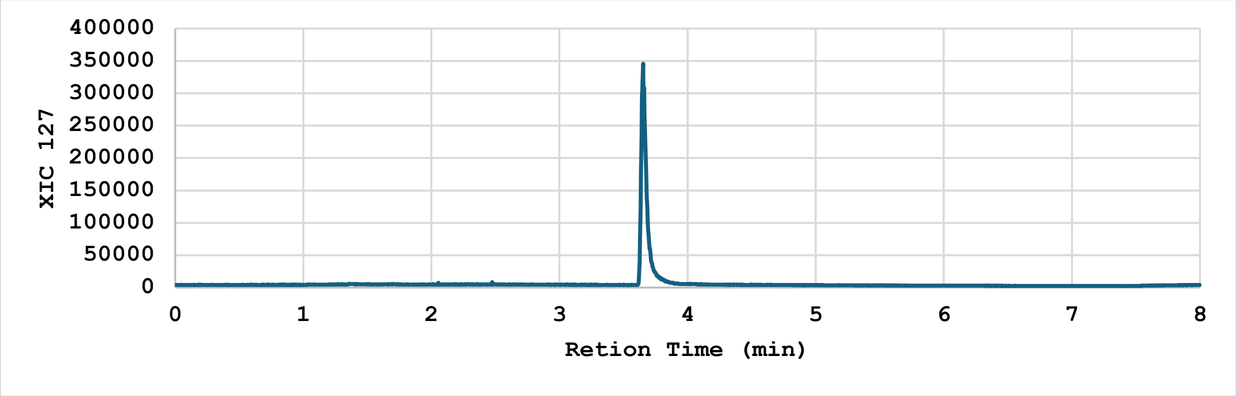

(B) XIC 625 µM TAL

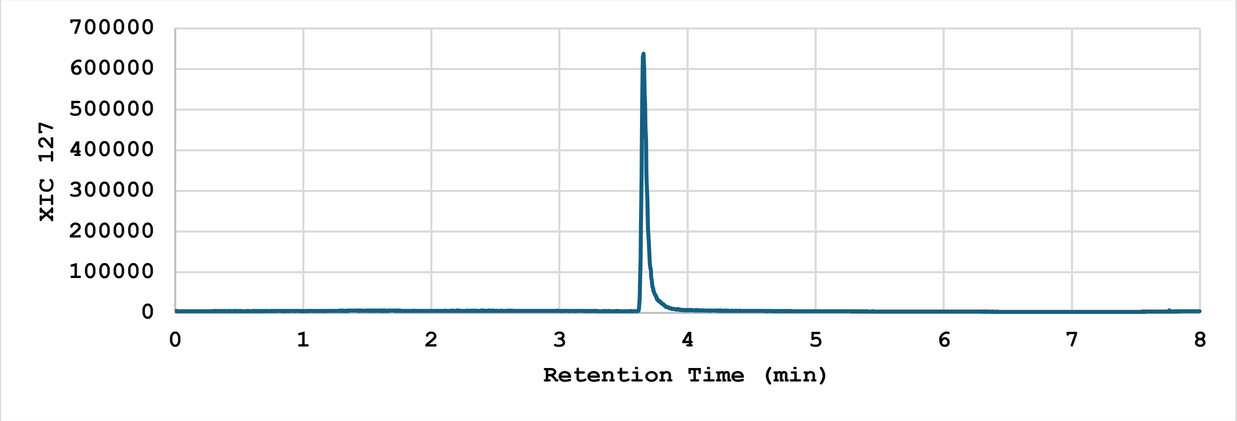

(C) XIC 1,222.5 µM TAL

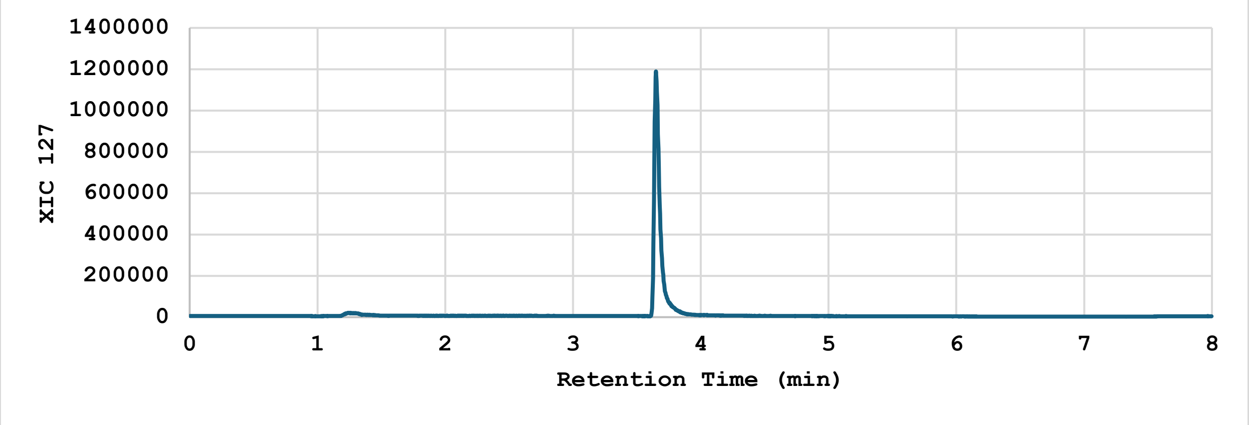

(D) XIC 4mM SNAC *in situ* reaction

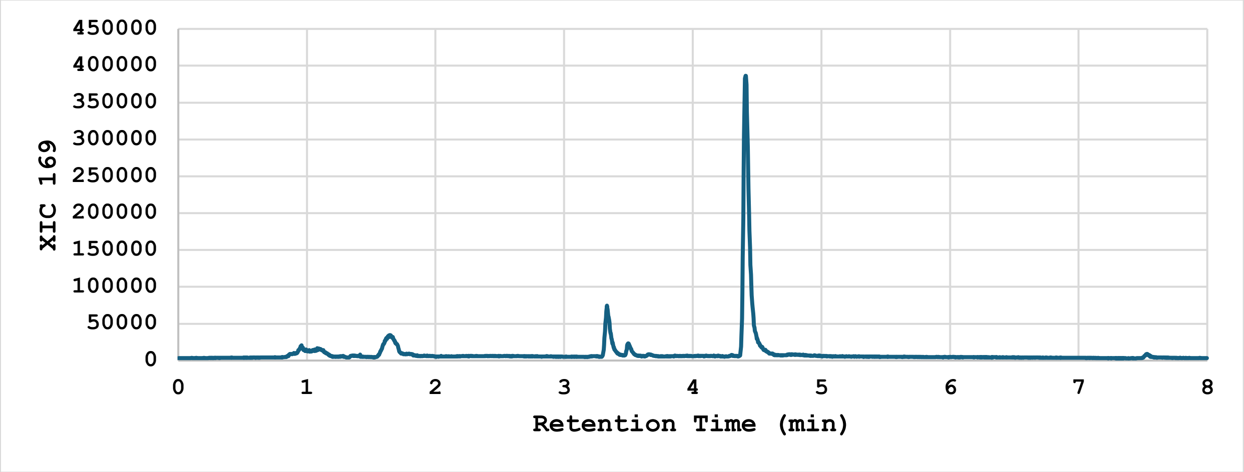

(E) Calibration curve used in determining pyrone concentration

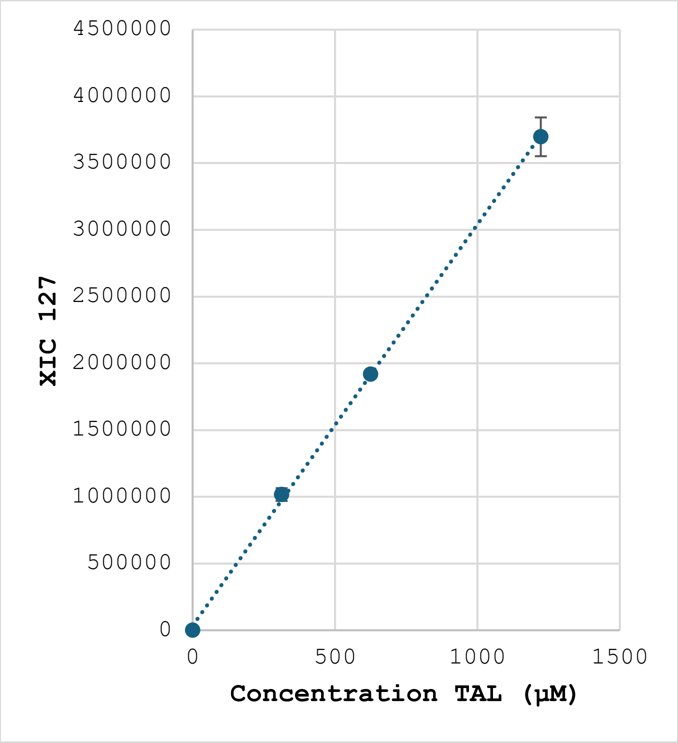

Supplementary Figure S8. Scheme showing a mechanism for pyrone formation via EryA1TE. The mechanistic steps are abbreviated. Stereochemistry is not shown for brevity.

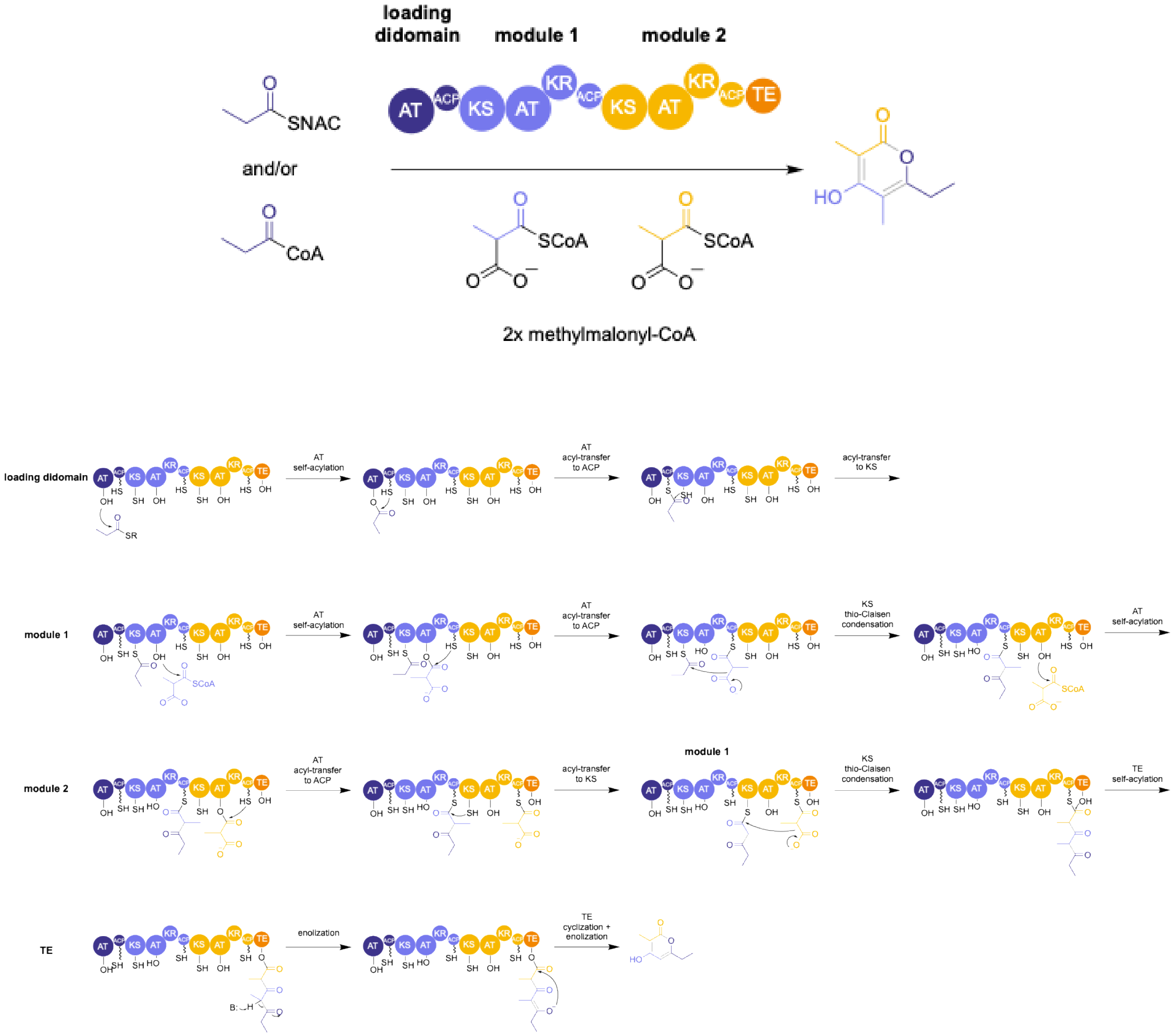

Supplemental Methods

HPLC Analysis of Quenched AcsA Catalyzed Reactions

After remaining at -20 ˚C overnight, quenched enzyme was removed via centrifugation at 21,000 g's for 1 hour. The supernatant was then aliquoted into 100 µL vial inserts. Subsequently, these reactions were analyzed on a Shimadzu HPLC using the linear gradient described below.

A: 0.1% TFA in water
B: Methanol

Step	Gradient/ % B	Method Time
1	0% - 40% B	0 – 29 min
2	100%	29 – 32 min
3	0% B	32 – 35 min

Samples were monitored using a D_2 lamp measuring absorbance from 190 nm to 900 nm.

LCMS Analysis of quenched AcsA Catalyzed Reactions

After remaining at -20 ˚C overnight, quenched enzyme was removed via centrifugation at 21,000 g's for 1 hour. 20 µL of the remaining supernatant was then diluted into a fresh 80 µL of chilled MEOH. After light vortexing the diluted reactions were left for 1 hour at -20 ˚C. Subsequently, these reactions were centrifuged again at 21,000 g's for 1 hour and then aliquoted into 100 µL spring vial inserts. Reactions were then analyzed on a Shimadzu LCMS2050 using the method described below.

LCMS: Shimadzu LCMS-2050 equipped with PDA (SPD-M40)
Column: Shimadzu Nexcol 5 µm C18 50 x 3.0 mm
Linear gradient -
A: 0.1% formic in water
B: 0.1% formic acid in acetonitrile
0 – 5 min: 0 - 99% B
5 – 5.5 min: 99% B
5.5 – 7 min: 0% B

Model Generation of AcsA$_{Pc}$

A AcsA$_{Pc}$ homology model was created using I-Tasser.[1–3] The confidence in the model is represented by a C-value. C-values range between -5 and 2 with high C-values representing higher confidence. The model used in this study had a C value of 0.94.

Simultaneously, an AlphaFold generated model of AcsA$_{Pc}$ was produced with an iPTM score of 0.94 where a PTM value greater than 0.8 represents "confident high-quality predictions". Alpha fold models of each of the high performing mutants were generated similarly and found to have similar iPTM scores.

Docking Study AcsA$_{Pc}$

Docking studies for each of the Thiols of interest in WT AcsA were conducted using the DockingPy plugin associated with Pymol.

Saturation and Random Mutagenesis of AcsA

Error-prone PCR was performed using a Genemorph II kit (Agilent) in a total volume of 50 uL using 500 ng of pET-28a/AcsA as a template, 0.8 mM dNTPs, 0.5 uL Mutazyme II and primers 1 and 2 (Table S4). The backbone of pET-28a (+) was amplified using the standard Phusion protocol and primers 3 and 4 (Table S4). The two pieces were digested with restriction enzymes *Hin*dIII and *Bam*HI for 1 h and 15 min at 37 °C. The products of the restriction digests were separated on a 0.1% agarose gel and 140 mV, where they were then gel extracted using an NEB Gel extraction kit. 100 uL of each isolated fragment was then ligated using an NEB T4 DNA ligase per the manufacturer's recommendations. The ligation mixture was transformed into *E. coli* Top10 chemically competent cells and grown for 1 h. The culture was plated across LB agar plates supplemented with 30 μg/mL kanamycin.

The following day, 10 ml cultures supplemented with 30 μg/mL kanamycin were grown for five colonies on the plate to confirm the success of the PCR and to determine the mutation rate. Once the success was confirmed, the plates were washed with 1x protein buffer saline (PBS), and the DNA was extracted using the standard miniprep protocol and re-transformed into *E. coli* BL21(DE3).

Site-directed saturation mutagenesis was performed using round-the-horn amplification using two NNK primers (per library). Refer to Table S4 for each specific site’s primers. The amplified fragments were run on a 0.1% agarose gel at 140 mV, where their relative size compared to a standard ladder was confirmed. The linear DNA fragment was transformed into Top10 chemically competent cells and grown for 1 h. The culture was plated across LB agar plates supplemented with 30 μg/mL kanamycin.

The remaining steps in the library construction follow those listed above for the random mutant library.

Synthesis of HSNAC

HSNAC was synthesized in accordance with our previous works[4]

Testing of Initial Reaction Rates

A premixed solution of sodium phosphate buffer (200 mM, pH 7) containing MgCl2 (2 mM), (NH4)2SO4 (100 mM), ATP (0.4 mM), thiol of interest (0.2 mM), and propanoic acid (0.4 mM) was made and distributed in 500 µL aliquots. Reactions were initiated via addition of purified AcsA$_{Pc}$ enzyme to a final concentration of 0.05 µg/ µL and left to react at RT. Reactions were then quenched at set times of 2 min, 5 min, 10 min, 20 min, 40 min, and 60 min by removing 50 µL aliquots and quenching them into an equal volume of ice-cold methanol and chilled at −20 °C overnight. The reactions were then analyzed via LCMS (SNAC reactions) and HPLC (CoA reactions) as done to the 1 h end point assays.

Acid Panel Testing

A premixed solution of sodium phosphate buffer (200 mM, pH 7) containing MgCl2 (2 mM), (NH4)2SO4 (100 mM), ATP (0.4 mM), thiol of interest (0.2 mM), and acid of interest (0.4 mM) was made and distributed in 100 µL aliquots. Reactions were initiated by adding purified $AcsA_{Pc}$ enzyme to a final concentration of 0.05 µg/µL and left to react at RT on the benchtop for 1 h. Reactions were then quenched by adding an equal volume of ice-cold methanol and chilled at −20 °C overnight, then subsequently analyzed via HPLC (CoA reactions) or LCMS (SNAC reactions).

1. Zhang, Y. (2008) I-TASSER server for protein 3D structure prediction. *BMC Bioinformatics*, **9** (1), 40.
2. Roy, A., Kucukural, A., and Zhang, Y. (2010) I-TASSER: a unified platform for automated protein structure and function prediction. *Nat. Protoc.*, **5** (4), 725–738.
3. Yang, J., Yan, R., Roy, A., Xu, D., Poisson, J., and Zhang, Y. (2015) The I-TASSER Suite: protein structure and function prediction. *Nat. Methods*, **12** (1), 7–8.
4. Cossin, J.R., Paulsel, T.Q., Castelli, K., Wcisel, B., Malico, A.A., and Williams, G.J. (2025) Engineering the Specificity of Acetyl-CoA Synthetase for Diverse Acyl-CoA Thioester Generation. *ACS Chem. Biol.*, **20** (4), 930–941.
